# LDL atherogenicity determined by size, density, oxidation, apolipoprotein(a), and electronegativity: an updated review

**DOI:** 10.3389/fcvm.2025.1649759

**Published:** 2025-10-24

**Authors:** Omer Akyol, Huan-Hsing Chiang, Alan R. Burns, Chao-Yuh Yang, Darren G. Woodside, Tatsuya Sawamura, José Luis Sánchez-Quesada, Antonio M. Gotto, Chu-Huang Chen

**Affiliations:** ^1^ Molecular Cardiology Research Laboratories, Vascular and Medicinal Research, The Texas Heart Institute at Baylor College of Medicine, Houston, TX, United States; ^2^ Biological Imaging Core, The University of Houston College of Optometry, Houston, TX, United States; ^3^ Department of Medicine, Baylor College of Medicine, Houston, TX, United States; ^4^ Molecular Cardiology Research Laboratories, The Texas Heart Institute at Baylor College of Medicine, Houston, TX, United States; ^5^ Department of Molecular Pathophysiology, Shinshu University School of Medicine, Matsumoto, Japan; ^6^ Cardiovascular Biochemistry, Institut de Recerca Sant Pau (IR-Sant Pau), Barcelona, Spain; ^7^ CIBER of Diabetes and Metabolic Diseases (CIBERDEM), Barcelona, Spain; ^8^ Cardiac Disease Prevention, Weill Cornell Medical College, New York, NY, United States

**Keywords:** atherosclerosis, atherosclerotic cardiovascular disease (ASCVD), small, dense LDL (sdLDL), oxidized LDL(oxLDL), lipoprotein(a) [Lp(a)], electronegative LDL [L5/LDL(-)]

## Abstract

Atherosclerotic cardiovascular disease (ASCVD), including coronary heart disease and cerebrovascular disease, is caused by the accumulation of plaque on artery walls. Elevated levels of low-density lipoprotein (LDL) cholesterol significantly contribute to the development and progression of ASCVD. Multiple studies have provided evidence of a correlation between individual LDL subpopulations and the development of atherosclerosis (AS); among these, small, dense low-density lipoprotein (sdLDL) and lipoprotein(a) [Lp(a)] have been particularly implicated. There are multiple considerations of why sdLDL may cause AS including their low affinity for the LDL receptor, their ability to diffuse into the artery wall and remain there for a long time, and their tendency to become excessively oxidized. Oxidized LDL (oxLDL), generated under oxidative stress, drives AS by impairing endothelial function, promoting foam cell formation, and triggering vascular inflammation. Lp(a) contributes to the development and progression of AS by causing inflammation of the arterial wall. Studies conducted in recent years have found that electronegative LDL [L5/LDL(-)] may also be an important factor in the development and progression of AS. L5/LDL(-) causes atherosclerotic changes in the vascular wall by triggering apoptosis in endothelial cells via the lectin-like oxLDL receptor-1. This article offers an updated overview of ASCVD and briefly examines the classifications of atherogenic LDL subfractions and their roles in atherogenesis.

## 1 Introduction

Extensive evidence has consistently substantiated the pivotal role of low-density lipoprotein (LDL) in both the initiation and advancement of atherosclerotic cardiovascular disease (ASCVD) (1–4). Consequently, the measurement of LDL levels is paramount in the comprehensive evaluation of cardiovascular risk, as emphasized in universally recognized international guidelines (5, 6).

Lowering LDL-C remains the cornerstone of ASCVD prevention and treatment. However, clinical observations reveal a paradox: some individuals with moderately elevated LDL-C remain free of ASCVD, while others with low LDL-C still experience major cardiovascular events such as ST-elevation myocardial infarction (STEMI) and stroke (7, 8). These discrepancies highlight the need to look beyond total LDL-C levels to identify the truly atherogenic subfractions or variants of LDL, including small, dense LDL (sdLDL), oxidized LDL (oxLDL), lipoprotein(a) [Lp(a)], and electronegative LDL [L5/LDL(-)] (9). While LDL-C levels and subfraction characterization provide valuable insights into atherogenic processes, they do not fully account for the residual cardiovascular risk that persists despite optimal lipid-lowering therapy (10). Additional factors such as inflammation, elevated Lp(a), triglyceride (TG)-rich lipoproteins, and non-lipid contributors play important roles in ASCVD pathogenesis, underscoring that the study of LDL subfraction should be integrated into a broader, multifactorial risk assessment strategy (11).

Several research studies have provided evidence demonstrating a correlation between distinct subgroups of LDL and the occurrence of atherosclerosis (AS). Specifically, sdLDL has been identified as not only closely related to AS but also exerting a more pronounced impact (12). oxLDL is toxic toward endothelial cells (ECs), smooth muscle cells (SMCs), and fibroblasts; proliferating cells are more susceptible than quiescent cells in the S-phase of the cell cycle, i.e., during DNA synthesis (13). Lp(a) has been thought to encompass a spectrum of potential deleterious effects, including pro-atherosclerotic, prothrombotic, and pro-inflammatory roles (14). L5/LDL(-), on the other hand, has been established as a dynamically active participant in the atherosclerotic process and is considered a potential inflammatory biomarker (15). The purpose of this review is to investigate the interrelationship between sdLDL, oxLDL, Lp(a), and L5/LDL(-) levels and their association with AS.

## 2 Current atherogenic LDL classes and their physicochemical characteristics

LDL is generally characterized as a lipoprotein fraction with a density ranging from 1.019 to 1.063 g/ml, which can be separated using different laboratory techniques (16). The LDL fraction is not uniform and can be divided based on physicochemical properties such as density, size, and composition, with specific subfractions like sdLDL, oxLDL, Lp(a), and L5/LDL(-) linked to an increased risk of ASCVD.

### 2.1 sdLDL

It is a subfraction of LDL characterized by its smaller size and higher density, which is highly associated with increased risk of AS and cardiovascular disease (CVD) due to its ability to penetrate the arterial wall and their high susceptibility to modification. Ultracentrifugation was employed to categorize LDL (Table 1) into four types based on density: LDL I (large): 1.019–1.023 g/ml, LDL II (intermediate): 1.023–1.034 g/ml; LDL III (small): 1.034–1.044 g/ml; LDL IV (very small): 1.044–1.063 g/ml (17). Another technique extensively utilized for the identification of LDL is gradient gel electrophoresis (GGE) (Table 1), whereby LDL particles are separated by electrophoretic mobility, primarily determined by particle sizes (18). sdLDL, collectively referred to as LDL particles with densities >1.034 g/ml and a diameter <25.5 nm measured by GGE, are formed when plasma TG levels are elevated. Conversely, the observation of larger, more buoyant, or medium-sized LDLs with densities ≤1.034 g/ml and diameters ≥25.5 nm occurs at lower TG levels (19). Synthesis of sdLDL involves several complicated steps, not clearly understood yet. The generation of TG-rich very low-density lipoprotein (VLDL) large particles is induced by hypertriglyceridemia. Initially, TG-rich VLDL undergoes hydrolysis facilitated by lipoprotein lipase (LPL), followed by cholesterol ester (CE) transfer protein (CETP)-mediated exchange between TGs from VLDL and CEs from LDL and high-density lipoprotein (HDL) particles, leading to the formation of TG-rich LDLs. Subsequently, these LDLs are delipidated by hepatic lipase (HL) and transformed into smaller, denser (sdLDL) forms (20). The efficiency and extent of sdLDL formation vary considerably among individuals, largely due to metabolic factors such as insulin resistance. These factors alter lipoprotein metabolism and modulate HL activity, which in turn regulates TG hydrolysis and LDL remodeling (21). These modulators play essential roles in determining sdLDL concentrations and their atherogenic potential across different patient populations.

**Table 1:** Methodologies^a^ for evaluating LDL particle density, size, and electronegativity.

Methodology	Parameter measured	Description	Advantages	Limitations
Ultracentrifugation KBr gradient	LDL density	Separates LDL based on density using high-speed centrifugation.	Gold standard for density separation; highly reproducible.	Time-consuming; requires specialized equipment; does not measure size directly.
Ultracentrifugation D_2_O/sucrose gradient	LDL density	Separates LDL based on density using high-speed centrifugation.	Low-ionic strength maintains LDL in native state. Do not require subsequent dialysis.	Time-consuming; requires specialized equipment; expensive; does not measure size directly.
Iodixanol gradient ultracentrifugation	LDL density	Separates LDL based on its buoyant density in a non-ionic, iso-osmotic medium	Maintains LDL integrity due to iso-osmotic and non-toxic conditions; high resolution; minimizes protein denaturation	Time-consuming and labor-intensive; requires specialized equipment; limited scalability and throughput for large sample numbers
Gradient gel electrophoresis	LDL size	Separates LDL particles by size using polyacrylamide gels.	Provides detailed size distribution; relatively simple.	Limited resolution; may not distinguish closely related sizes.
Agarose gel electrophoresis	LDL electronegativity	Separates LDL particles based on charge using agarose gels.	Simple and cost-effective; useful for assessing electronegativity.	Limited resolution; qualitative rather than quantitative.
Anion exchange fast protein liquid chromatography (FPLC) after ultracentrifugation^b^	LDL electronegativity	Separates LDL particles based on charge using anion exchange column	Simple and cost-effective; useful for assessing electronegativity percentage	Semi-quantitative; requires specialized expertise
Gel filtration chromatography (SEC)	LDL size and relative abundance of LDL subfractions	Separates LDL particles based on hydrodynamic size by passing them through porous gel matrix.	Non-destructive, no need for harsh solvents, compatible with other detectors	Indirect density measurement (from size; sdLDL is denser), limited resolution.
Nuclear magnetic resonance (NMR) spectroscopy	LDL size and particle number	Measures the magnetic properties of LDL particles to determine size and concentration.	High throughput; provides detailed particle number and size data.	Expensive equipment requires specialized expertise.
Dynamic light scattering (DLS)	LDL size	Measures particle size based on light scattering fluctuations in solution.	Rapid and non-destructive; requires minimal sample preparation.	Less accurate for polydisperse samples; sensitive to contaminants.
Electron microscopy	LDL size and morphology	Visualizes LDL particles directly using electron beams.	Provides direct visualization of size and shape.	Expensive; time-consuming; requires specialized sample preparation.
Ion mobility analysis	LDL size distribution and collusion cross-section	Separates LDL particles based on their size, shape and charge in a gas phase under an electric field.	High resolution; can measure size and density simultaneously.	Expensive; limited availability.
Capillary isotachophoresis (cITP)	LDL size and charge	Separates LDL by their differential migration in an electric field, forming discrete zones.	High resolution; no need pre-staining or ultracentrifugation; fast and requires small sample volumes	Limited standardization across labs; sensitivity to buffer/pH conditions; less common
Heparin-Mg precipitation	Total LDL	Separates LDL from other lipoproteins including VLDL and HDL	Simple and quick; large-scale lipoprotein preparation	Overestimation of HDL-C; interferences from high TG

LDL Density: Refers to the buoyant density of LDL particles, which correlates with their lipid and protein composition.

LDL Size: Refers to the diameter of LDL particles, with smaller, denser LDL particles being more atherogenic.

LDL Electronegativity: Refers to the negative charge on LDL particles, which can increase due to oxidation or glycation, making them more atherogenic.

^a^ Each methodology has its strengths and limitations, and the choice of methods depends on the specific research or clinical question being addressed.

^b^ All teams investigating electronegative LDL employ this preparative FPLC isolation method, with only minor variations in processing, such as using gradual vs. stepwise gradients.

### 2.2 oxLDL

It is a modified form of LDL generated under oxidative stress conditions and is not a typical component of normal lipid metabolism. Rather, oxLDL acts as a pathological entity that promotes endothelial dysfunction, inflammation, and foam cell formation, thereby playing a central role in the development and progression of ASCVD. The oxidative modification of LDL occurs primarily in the subendothelial space, where it can interact with reactive oxygen species (ROS), enzymes, and metal ions (22). LDL oxidation takes place in a stepwise fashion. In the initial phase, oxidation begins with ROS, such as superoxide or hydroxyl radicals, extracting hydrogen atoms from polyunsaturated fatty acids (PUFAs) present in LDL phospholipids or CEs (23). The process forms lipid radicals, which react with oxygen to generate lipid peroxides. In the propagation phase, lipid peroxides undergo decomposition to form reactive aldehydes, such as malondialdehyde (MDA) and 4-hydroxy-2-nonenal (HNE). These aldehydes can covalently bind to the amino groups of Lys and Arg residues in apolipoprotein B100 (apoB-100), creating Schiff base adducts and cross-links (24). In the termination phase, the oxidation process is terminated when antioxidants, such as *α*-tocopherol, neutralize free radicals, or when oxLDL is cleared by scavenger receptors.

From the oxidative modifications, distinct structural and chemical changes in both the lipid and protein components of LDL occur (25). Phosphatidylcholine and other surface phospholipids are oxidized, reducing structure fluidity and altering particle stability. Cholesterol moieties are also prone to oxidation, leading to the formation of oxysterols such as 7-ketocholesterol, which further destabilizes the LDL particle (26). As for protein modifications, reactive aldehydes, like MDA and HNE, form covalent adducts with lysine residues of apoB-100, altering its structure and receptor-binding properties (27). Schiff base adducts on oxLDL impair its ability to be recognized by the LDL receptor (LDLR), instead promoting uptake by scavenger receptors.

The fate of oxLDL in the body is markedly different from that of native LDL due to its altered structure and receptor affinity. Native LDL is primarily cleared by the liver through interaction with LDLR. In contrast, oxLDL is not efficiently recognized by the LDLR and is instead internalized by macrophages via scavenger receptors, including lectin-like oxidized-LDL receptor-1 (LOX-1), scavenger receptor class A (SR-A), and CD36 (28). This receptor-mediated uptake leads to accumulation of cholesterol within macrophages, forming foam cells. ECs, SMCs, and even hepatocytes can interact with oxLDL through various receptors in terms of degradation of these molecules. The altered structure of oxLDL can lead to impaired cellular processes, including changes in lipid metabolism, signal transduction, and gene expression.

### 2.3 Lp(a)

As a variant of LDL, Lp(a) stands as a well-established risk factor for AS, coronary artery disease (CAD), stroke, thrombosis, and aortic stenosis, with its association being genetically determined (29). Structurally, Lp(a) is characterized by the presence of apolipoprotein(a) [apo(a)] bound to apoB-100 with a single disulfide bridge through sulfhydryl group binding and noncovalent interactions with lysine moieties. Lp(a) particles have a spherical structure (24 nm–28.3 nm diameter, density 1.050 g/ml–1.101 g/ml) with apo(a) causing density and mobility differences compared to LDL. Human Lp(a) varies in size and density due to an apo(a) polymorphism in the *APOA* gene (30).

Lp(a) functions as a major carrier of oxidized phospholipids (oxPL) in human plasma, influencing events related to atherothrombotic CVD and calcific aortic valve injury (31). Lp(a) is sensitive to environmental changes *in vivo*, such as alterations in salt concentrations, impacting its diameter and external characteristics. Electron microscopy studies of oxidized Lp(a) [oxLp(a)] reveal structural changes associated with oxidation. These alterations may underlie the observed reduction in lipid extractability, possibly reflecting tighter lipid binding or protein crosslinking (31). These changes may affect oxLp(a) recognition by LDLR and scavenger receptors, influencing the formation of foam cells and the accumulation of lipids, especially cholesterol, in vessel walls.

### 2.4 Electronegative LDL [L5/LDL(-)]

In literature, different research groups describe electronegative LDL in various ways. Some groups simplify the terminology by collectively referring to all negatively charged components of LDL as LDL(-), grouping them under a single category (32, 33). However, our definition is somewhat more nuanced. Following fast protein liquid chromatography (FPLC) fractionation of LDL, the least electronegative form is designated as L1, while the most electronegative form is categorized as L5 (34). Our definition differs because our research has shown that the lipid and apolipoprotein composition of L5 is significantly distinct from that of L1 (34, 35). Therefore, the characterization of L5 is not based solely on electronegativity but also considers other structural differences. To prevent any confusion for readers of this article, it should be clarified that, throughout the manuscript, we refer to these particles uniformly as L5/LDL(-), to standardize terminology and ensuring consistency.

Distinct physicochemical properties, which markedly differentiate it from conventional LDL, characterize L5/LDL(-). Relative to native LDL, L5/LDL(-) displays an altered lipid composition characterized by elevated levels of TGs, non-esterified FAs, ceramide, and lysophosphatidylcholine (LPC) (36). These compositional changes confer a more pro-atherogenic and pro-inflammatory phenotype. Elevated TGs and non-esterified FAs can destabilize lipoprotein structure, enhance susceptibility to oxidative modification, and promote endothelial lipotoxicity (20). Increased ceramide content activates signaling pathways such as LOX-1-mediated NF-κB activation, leading to oxidative stress, inflammatory cytokine release, endothelial dysfunction, and apoptosis (37, 38). LPC further amplifies vascular inflammation by inducing adhesion molecule expressions such as vascular cell adhesion molecule-1 (VCAM-1) and intercellular adhesion molecule-1 (ICAM-1), facilitating monocyte recruitment and foam cell formation (39). Together, these alterations enhance L5/LDL(-)'s ability to penetrate the arterial wall, stimulate immune cell activation, impair nitric oxide (NO) bioavailability, and accelerate atherogenesis.

Additionally, L5/LDL(-) displays an abnormal conformation of the amino-terminal region of apoB-100 (40). L5/LDL(-) has been shown to possess phospholipolytic activity, which is typically absent in native LDL, and this enzymatic function contributes to its remodeling and enhanced propensity for self-aggregation (41). Notably, significant differences in CE levels have been observed between native LDL and L5/LDL(-) (34). However, compared to L5/LDL(-) from individuals with normal lipid levels, L5/LDL(-) from patients with familial hypercholesterolemia contains lower CE (34). Similar differences were also reported in patients with diabetes mellitus (DM) (35). These biochemical properties may underlie the pathobiological effects of L5/LDL(-) on various cell types involved in AS progression. Itabe et al. reported that L5/LDL(-) shows substantial reduction in phospholipid levels and an increase in free cholesterol levels (42). Reduced phospholipid levels in L5/LDL(-) may stem from oxidation, as oxPLs are more readily hydrolyzed by phospholipase enzymes compared to non-oxPL (43). However, the findings related to L5/LDL(-)'s reduced phospholipid content remain to be confirmed. Another relevant difference between native LDL and LDL(-)/L5 is an increased content of apolipoproteins other than apoB-100 in the latter. Among others, the content of apolipoprotein E (apoE), apolipoprotein C-III (apoC-III), apolipoprotein A-1 (apoA-I), apolipoprotein D (apoD), apolipoprotein J (apoJ, clusterin) or apolipoprotein F (apoF) is clearly increased in L5/LDL(-) (44, 45). The increased content of these apolipoproteins compared to native LDL may further modulate L5/LDL(-)'s atherogenic and inflammatory properties. Elevated apoE can enhance LDL binding to heparan sulfate proteoglycans in the arterial intima, facilitating retention, while also altering receptor-mediated uptake pathways (46, 47). ApoC-III is strongly pro-atherogenic, inhibiting LPL-mediated TG hydrolysis and hepatic clearance, thereby prolonging particle residence time in circulation (48). Although apoA-I is generally anti-atherogenic (49), its presence on L5/LDL(-) may reflect an exchange from HDL and potential functional impairment, limiting reverse cholesterol transport. ApoD and apoJ are associated with lipid remodeling and stress responses (50), but their enrichment on L5/LDL(-) could contribute to altered lipid trafficking and protection of damaged lipoproteins, potentially stabilizing a pro-inflammatory profile. Increased apoF, an inhibitor of CETP, may modify lipid exchange between lipoproteins, influencing particle composition and metabolism (51). Collectively, these apolipoprotein alterations may synergize with L5/LDL(–)'s abnormal lipid composition to promote vascular retention, impair clearance, and perpetuate vascular inflammation.

## 3 Methodologies for assessing LDL particle density, size, and electronegativity

There are several methodologies used for assessing LDL particle density, size, and electronegativity (Table 1).

Density gradient ultracentrifugation technique separates LDL particles into subfractions based on their density, typically divided into three to four subclasses. Another ultracentrifugation method, iodixanol gradient ultracentrifugation, is a variation that uses iodixanol instead of salt gradients, providing slightly different density ranges for LDL subfractions (52).

Gradient gel electrophoresis separates LDL particles based on size under non-denaturing conditions. LDL subclasses are typically defined as: LDL I (large): 26.4–28.5 nm; LDL II (intermediate): 25.5–26.4 nm; LDL III A and B (small): 24.2–25.5 nm; LDL IV A and B (very small): 22.0–24.2 nm (19). Two phenotypes are distinguished based on peak LDL particle diameters: Pattern A: >25.5 nm (large and intermediate LDL), Pattern B: ≤25.5 nm (small and very small LDL).

Nuclear magnetic resonance (NMR) spectroscopy is a laboratory technique used to directly measure LDL particle number and size distribution (53). By providing detailed information on lipoprotein particle size, concentration, and composition, NMR-derived lipoprotein profiles have become a valuable adjunct to cardiovascular risk assessment. Precipitation methods separate sdLDL from larger LDL particles using different reagents (54, 55). Specifically, heparin-magnesium precipitation separates sdLDL using detergent and sphingomyelinase treatment (56). Dynamic light scattering measures LDL particle size and can be used to assess LDL aggregation susceptibility (56). Anion exchange chromatography is used to separate LDL according to its electronegativity which separates L5/LDL(-) from native LDL (57). As a high-resolution electrophoretic technique, capillary isotachophoresis can also be used to assess LDL electronegativity (58).

Some studies combine these techniques to comprehensively evaluate LDL properties, including particle density, size, and electronegativity (59). These approaches often integrate ApoB measurements to estimate LDL particle number alongside TG levels for assessing particle size. While direct LDL-C quantification methods exist, they are less commonly used due to higher costs, leading many studies to rely on indirect estimations like the Friedewald equation (60). Importantly, these analytical methods focus on LDL characterization for their atherogenic potential and should not be confused with the clinical concept of the LDL Window, which refers to the duration of LDL reduction after apheresis therapy.

## 4 Density distribution, size variability, and atherogenic potential of LDL particles

The size variability and density distribution of LDL particles plays a crucial role in determining their atherogenic potential, and understanding this heterogeneity is key to elucidating the complex mechanisms underlying CVD. As mentioned, LDL particles are not uniform; they exhibit a broad range of sizes and densities that influence their biological behavior, metabolism, and interaction with the arterial wall. This variability has significant clinical implications, as different LDL subfractions contribute to AS in distinct ways.

sdLDL particles are often associated with a greater atherogenic risk compared to larger, buoyant LDL particles due to several mechanistic properties, such as a greater propensity for arterial wall retention and increased susceptibility to oxidation (61). Increased proportion of sdLDL is associated with higher cardiovascular risk, even when total LDL-C levels are within normal range (62). The presence of sdLDL is often part of an atherogenic lipoprotein phenotype, which includes elevated TGs and low HDL cholesterol (HDL-C) (63). Studies have demonstrated that sdLDL is an independent risk factor for CVD, emphasizing the importance of both LDL quantity and quality in assessing cardiovascular risk (64). It is important to note that while this density-based classification is widely used, some recent studies have challenged the notion that all large LDL particles are less atherogenic. For instance, one study found that both very small and very large LDL particle sizes were associated with increased mortality risk compared to intermediate-sized LDL (63). sdLDL particles are also distinguished by their longer plasma residence time due to a reduced affinity for LDLR, which decreases their clearance from the circulation. This prolonged exposure time allows for increased interaction with arterial proteoglycans, promoting their retention and subsequent uptake by macrophages (1). Furthermore, these particles exhibit altered lipid and protein composition, such as a higher ratio of TG to CEs, which may render them more prone to oxidation, desialylation, and glycation modifications, thereby enhancing their atherogenic potential (65).

One of the primary characteristics that enhances the atherogenicity of sdLDL particles is their increased ability to penetrate the endothelial barrier and accumulate in the subendothelial space (66). Once trapped, these particles are more likely to undergo oxidative modification, forming oxLDL, which is a potent pro-inflammatory agent and a key contributor to plaque formation and instability. oxLDL is recognized by scavenger receptors on macrophages, leading to the formation of foam cells and the initiation of an inflammatory cascade within the arterial wall (67). This sequence of events is a critical step in the development and progression of atherosclerotic plaques, highlighting the importance of LDL particle size in influencing disease outcomes.

The relationship between LDL particle size and cardiovascular risk has been substantiated by several epidemiological and clinical studies. Individuals with a predominance of sdLDL particles are more likely to exhibit atherogenic dyslipidemia, characterized by elevated TGs, low HDL-C, and an increased number of LDL particles. This pattern, often observed in patients with metabolic syndrome and type 2 DM (T2DM), is associated with a higher risk of CAD and other cardiovascular events (CVEs) (68). Recent meta-analyses confirm sdLDL particles are independently associated with an increased risk of CVEs, even after adjusting for traditional risk factors such as total LDL-C levels (64). As mentioned earlier, advanced lipid testing methods, such as NMR spectroscopy and ion mobility analysis, have enabled more precise quantification and characterization of LDL subfractions. These technologies allow for the identification of sdLDL particles and provide valuable information on their concentration and distribution within the LDL particle spectrum. Incorporating these measurements into routine clinical practice could improve risk stratification and enable more targeted therapeutic strategies for managing dyslipidemia and preventing CVD (69). However, due to the substantial discrepancies between NMR-derived sdLDL concentrations and those obtained via traditional methods such as ultracentrifugation or GGE, with NMR generally yielding higher values, it is necessary to carefully calibrate or validate NMR methodologies to ensure comparability across platforms.

Given the distinct atherogenic profile of sdLDL particles, emerging therapeutic approaches are focusing on not just lowering overall LDL-C levels but also modifying the distribution of LDL subfractions. Interventions that increase the size of LDL particles, such as the use of fibrates or omega-3 FAs, have been shown to reduce cardiovascular risk, particularly in patients with a predominance of sdLDL (70). Similarly, lifestyle interventions such as dietary modifications and physical activity can significantly impact LDL particle size and density, highlighting the importance of a holistic approach to cardiovascular risk management (71).

Lp(a) levels, largely determined by genetics, show significant interindividual variability, ranging from <1 mg/dl to >1,000 mg/dl in the general population and remain stable throughout life. Elevated Lp(a) levels are linked to inflammatory conditions such as rheumatoid arthritis (RA) (72) and systemic lupus erythematosus (73). The atherogenic potential of Lp(a) is influenced by its structural properties and interactions with the vasculature. Lp(a) particles consist of an LDL core covalently bound to apo(a), which exhibits extensive size polymorphism. This variability arises from the number of kringle IV type 2 repeats in apo(a), affecting both particle size and plasma concentrations. Smaller apo(a) isoforms are associated with higher Lp(a) levels and increased atherogenicity. Lp(a) promotes atherogenesis through mechanisms including direct deposition onto arterial walls, facilitated by its greater susceptibility to oxidation compared to LDL (74). In fact, Lp(a) is the main transporter of oxPL in blood (75). Oxidized Lp(a) is readily taken up by macrophages via scavenger receptors, leading to foam cell formation, a key step in AS development. Additionally, elevated Lp(a) levels correlate inversely with vascular reactivity, contributing to endothelial dysfunction (76).

In addition to classifying LDL subfractions by size, density, or electronegativity, recent studies have emphasized the importance of quantifying particle mass composition, namely, the relative content of TG, free and esterified cholesterol, phospholipids, and protein within each subclass (77). These compositional profiles can vary significantly between LDL subfractions and are influenced by underlying metabolic states. For example, TG-enriched LDL particles are often found in insulin-resistant individuals and may correlate with delayed hepatic clearance and increased atherogenicity (78, 79). Advanced lipidomic approaches now allow for more precise characterization of these features, offering the potential to transform LDL subclass analysis from a descriptive tool into a quantitative, decision-support metric (80, 81). Incorporating compositional data may enhance cardiovascular risk stratification and support more individualized lipid-lowering strategies.

## 5 Electronegativity of LDL: mechanisms and implications

Electronegativity of LDL is a key factor in its atherogenic potential, with more electronegative LDL particles showing increased propensity for promoting AS. The formation of L5/LDL(-) occurs through various mechanisms, including oxidation, glycation, desialylation, and enzymatic modifications (82). These processes alter both the lipid and protein components of LDL, leading to changes in density, particle size, and surface charge. Oxidation, in particular, plays a crucial role in increasing LDL electronegativity, with oxPLs and modified apoB-100 contributing to the increased negative charge (83). Glycation of LDL, which is prevalent in diabetic conditions, also enhances L5/LDL(-)'s electronegativity and atherogenicity (40). Desialylation has also been related with the formation of L5/LDL(-) particles (84). Other factors, such as an abnormal conformation of apoB-100 (85, 86), increased apolipoprotein content (44, 45), and especially non-esterified fatty acids (NEFA) (87, 88), are also key determinants in increasing the electronegativity of LDL particles. Finally, the fact that a significant proportion of L5/LDL(-) is made up of sdLDL (88, 89) also contributes to an increase in electronegativity, since sdLDL has a greater negative charge than large or intermediate LDL particles (90).

The increased negative charge of LDL particles affects their interactions with extracellular matrix components and cell surface receptors. For instance, L5/LDL(-) shows a higher affinity for proteoglycans in the arterial wall, promoting its retention and accumulation in the subendothelial space (91, 92). Furthermore, L5/LDL(-) is preferentially recognized by scavenger receptors rather than the classical LDLR, leading to increased uptake by macrophages and foam cell formation (93). The electronegativity of LDL also influences its susceptibility to further modifications and aggregation, with more electronegative particles being more prone to additional oxidative changes and aggregation (94). Interestingly, recent studies have shown that L5/LDL(-) particles possess enzymatic activities, including platelet-activating factor acetyl hydrolase (95), phospholipase C (41) or ceramidase activities (36), which may play a role in modulating their effects on vascular cells (40). These properties contribute to the enhanced atherogenicity of L5/LDL(-). The presence of L5/LDL(-) in circulation has been associated with various pathological conditions, including CVD, DM, metabolic syndrome, and RA (96–102). Measurement of LDL electronegativity has been proposed as a potential biomarker for assessing cardiovascular risk, complementing traditional lipid profile analyses (83, 103, 104). Understanding the mechanisms underlying LDL electronegativity and its implications in atherogenesis provides valuable insights for developing targeted therapeutic strategies to mitigate the harmful effects of these modified lipoproteins in CVDs.

## 6 Factors influencing LDL particle properties

The complex interplay of genetic, metabolic, and lifestyle factors influences the physical and chemical properties of LDL particles such as density, size, composition, and oxidative state, which can have profound implications for their metabolism, clearance, and role in cardiovascular health (105, 106). These factors collectively determine the structural and functional characteristics of LDL, which in turn impact its behavior in lipid metabolism and its role in health and disease.

One key factor is genetic variation, particularly in genes related to apolipoproteins, lipid metabolism, and LDLR. For instance, variations in the *APOB* gene can alter the structure of apoB-100, which is the primary protein component of LDL, affecting its receptor-binding affinity and clearance rate (107). Similarly, polymorphisms in the *LDLR* gene can influence the uptake and degradation of LDL particles, leading to alterations in their plasma concentrations and composition (108). Genetic determinants also play a key role in Lp(a) atherogenicity. The *LPA* gene exhibits size polymorphisms due to a variable number of kringle IV type 2 repeats, which define the apo(a) isoform size. Fewer repeats result in smaller isoforms, which are linked to elevated plasma Lp(a) concentrations and a higher risk of AS and CVEs (109).

Metabolic conditions, such as insulin resistance and DM, also modulate LDL properties by altering lipid exchange and modifying enzymatic activities. Insulin resistance often leads to increased TG content in LDL, forming TG-enriched LDL particles, which are more prone to lipolysis by HL. This process generates sdLDL particles that are more atherogenic (106). Additionally, glycation of LDL particles in the context of hyperglycemia further modifies their structure, making them more susceptible to oxidative damage and less efficiently cleared by the LDLR pathway (110). These changes in LDL composition and size are further compounded by enzymatic activity involving LPL and CETP, which mediate lipid exchange among lipoproteins, altering the distribution of CEs and TGs across LDL, VLDL, and HDL fractions.

Lifestyle factors, such as diet, physical activity, and smoking, also play a significant role in shaping LDL particle properties. Diets high in saturated fats and cholesterol increase the hepatic production of apoB-containing lipoproteins, leading to elevated concentrations of large, buoyant LDL particles (111). Conversely, high-carbohydrate diets and excessive alcohol consumption tend to promote the formation of sdLDL particles by increasing hepatic VLDL production and enhancing the activity of CETP (105). Regular physical activity can reduce the concentration of sdLDL by improving insulin sensitivity and lowering TG levels, thus promoting the formation of larger, less dense LDL particles. On the other hand, smoking has been shown to increase oxidative stress, which oxidizes LDL lipids and proteins, making the particles more atherogenic and less recognizable by the LDLR (112).

## 7 Contribution of LDL subfractions and particle characteristics to atherogenesis and their clinical relevance

**Figure 1:**
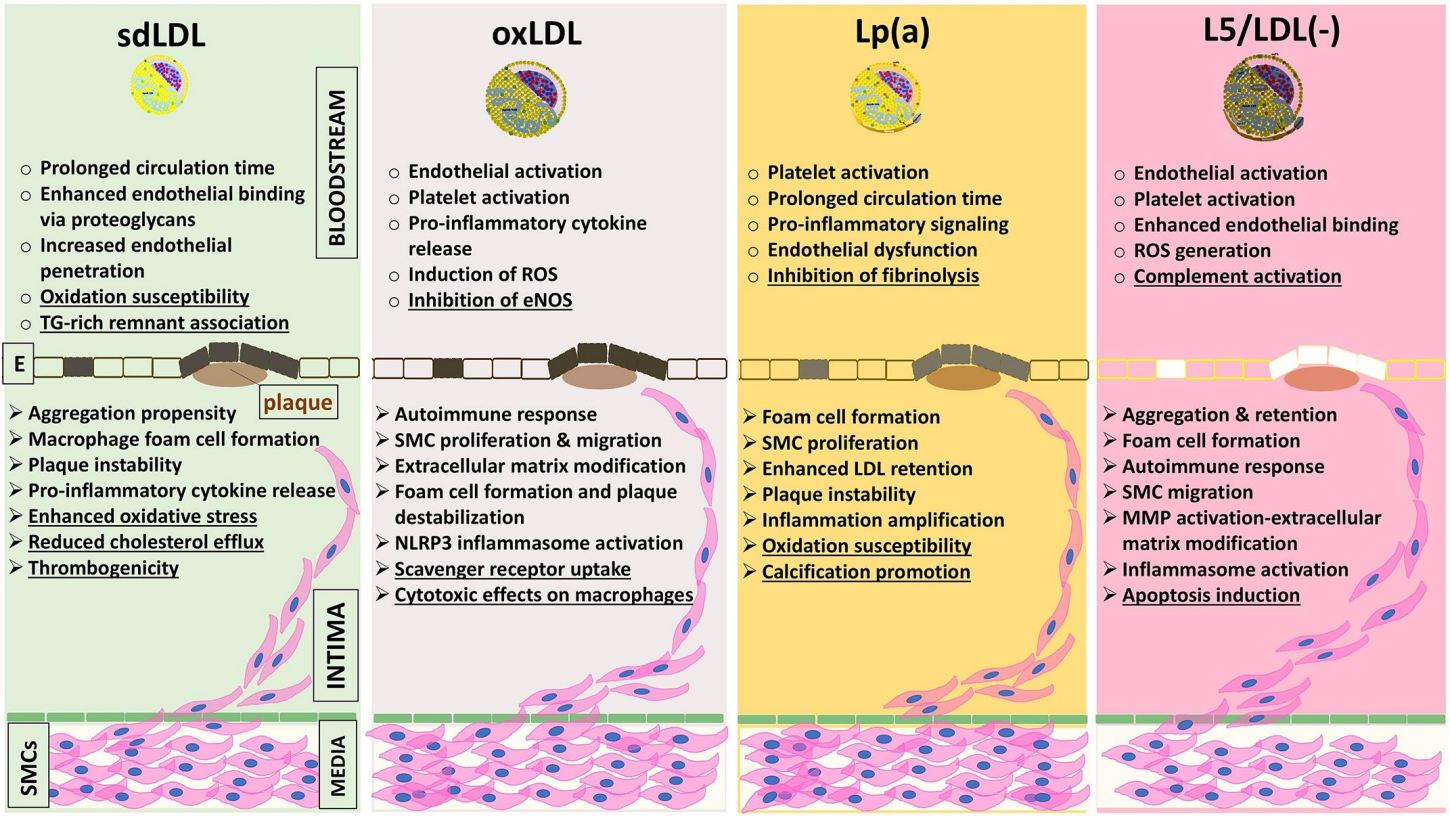
Schematic summary of the major pro-atherogenic mechanisms attributed to four pathogenic LDL subfractions, sdLDL (refs. 19–21, 61–69, 122–134), oxLDL (refs. 22, 23, 25, 28, 135–139), Lp(a) (refs. 29–31, 72–76, 140–145), and L5/LDL(-) (refs. 32–51, 146–150, 156, 172–175), organized by their actions in two anatomical compartments: the vessel lumen and the arterial intima. Dysfunctional endothelial cells (shaded between bloodstream and intima) mark the transition toward pathological changes. All four subfractions share key mechanisms, including reduced endothelial NO bioavailability, upregulation of adhesion molecules (VCAM-1, ICAM-1), proteoglycan binding [decorin, biglycan; relative affinity Lp(a)>L5/LDL(-) > sdLDL > oxLDL], macrophage cholesterol uptake (via CD36, LOX-1, SR-A1), activation of inflammatory pathways (NF-*κ*B, NLRP3 inflammasome, cytokines IL-1β and TNF-α), and plaque destabilization (MMP induction and vascular smooth muscle cell apoptosis). In addition, each subfraction displays unique features (underlined): sdLDL: oxidative susceptibility, reduced cholesterol efflux, thrombogenicity, and linkage to TG-rich remnants; oxLDL: eNOS inhibition, macrophage cytotoxicity, uptake via specialized scavenger receptors; Lp(a): antifibrinolytic effects via apolipoprotein(a), vascular calcification, and oxidative susceptibility; L5/LDL(-): complement activation and LOX-1–mediated apoptosis. CD36, scavenger receptor class B member 3; E, endothelium; eNOS, endothelial nitric oxide synthase; ICAM-1, intercellular adhesion molecule-1; IL-1β, interleukin-1 beta; L5/LDL(-), electronegative LDL; LDL, low-density lipoprotein; LOX-1, lectin-like oxidized LDL receptor-1; Lp(a), lipoprotein(a); MMPs, metalloproteinases; NF-κB, nuclear factor kappa B; NLRP3, nucleotide-binding domain, leucine-rich-containing family, pyrin domain-containing 3; NO, nitric oxide; oxLDL, oxidized LDL; ROS, reactive oxygen species; sdLDL, small dense LDL; SMC, smooth muscle cell; TG, triacylglycerol; TNF-α, tumor necrosis factor-α; VCAM-1, vascular cell adhesion molecule-1.

The clinical relevance of LDL particle characteristics in atherogenicity has become a focal point in cardiovascular research, emphasizing the importance of not just LDL-C levels, but also the properties of LDL particles themselves. Atherogenesis is a disorder of the artery wall characterized by various stages: initial adhesion of monocytes and lymphocytes to the EC surface, subsequent migration of these cells into the sub-endothelial space, and, in the case of monocytes, differentiation into macrophages. The proatherogenic effect of these LDL subfractions is primarily due to their enhanced arterial wall penetration, increased susceptibility to oxidative or enzymatic modification, and greater uptake by macrophages through scavenger receptors, which promotes foam cell formation (113). This leads to the accumulation of CEs and the formation of foam cells, which, along with T lymphocytes, contribute to the development of atheroma plaque (Figure 1). Additionally, vascular SMCs migrate from the media into the intima and proliferate, resulting in the formation of atherosclerotic plaques (31). The entire atherogenic process involves crucial cellular activities like adhesion, migration, differentiation, proliferation, and interactions with the extracellular matrix (114–117).

### 7.1 sdLDL

The enhanced atherogenic potential of sdLDL has been attributed to several proposed pathophysiologic mechanisms. As stated before, these particles exhibit an extended circulation time, likely due to a diminished affinity for the LDLR (19) and impaired clearance kinetics (118, 119). Under normal physiological conditions, apoB-containing lipoproteins undergo continuous remodeling during their metabolic transit, with LDL particles efficiently cleared via hepatic LDLR. However, in metabolically abnormal states such as insulin resistance, increased TG-rich lipoprotein exchange promotes the formation of sdLDL, which are more prone to oxidative and structural modifications. Due to their smaller size, sdLDL particles penetrate the arterial endothelium more readily than larger LDL (65) and are more susceptible to qualitative modifications such as oxidation, desialylation, and glycosylation (120). These changes can impair LDLR dimerization, a structural requirement for optimal ligand binding and internalization (121), thereby prolonging plasma half-life and promoting their accumulation in circulation. Such modifications also elicit an inflammatory response, increase particle affinity for intimal proteoglycans, enhance preferential uptake by macrophages, and contribute to foam cell formation (122). The greater propensity for uptake by arterial tissue of sdLDL compared to larger LDL has been reported (123), implying increased trans-endothelial transport of smaller particles. Moreover, smaller LDL particles may exhibit decreased receptor-mediated uptake and increased binding to proteoglycans (124–126).

*In vitro* studies have established that LDL subfractions vary in their susceptibility to oxidative stress, a critical factor in atherogenesis (127–130). This susceptibility is commonly assessed by measuring the lag time before the onset of lipid peroxidation during copper-induced oxidation. Specifically, large buoyant LDL exhibits greater resistance to oxidation, while sdLDL is more susceptible to due to its smaller size and higher PUFAs content (130, 131).

Individuals with a predominance of sdLDL particles are at heightened cardiovascular risk, in part due to their increased oxidative susceptibility and enhanced atherogenicity (62). Clinical studies have demonstrated that sdLDL particle number correlates strongly with CAD, metabolic syndrome, and T2DM (132, 133). Moreover, meta-analyses of prospective studies have shown that sdLDL levels are significantly associated with the progression of AS and CVEs. Multiple studies have consistently demonstrated that the cholesterol content of sdLDL (sdLDL-C) is a stronger predictor of ASCVD risk than total LDL-C or large, buoyant LDL cholesterol. Both direct measurements and estimation methods have shown that sdLDL-C levels more effectively discriminate ASCVD risk across diverse populations (12, 134–136). These findings highlight the clinical importance of assessing sdLDL-C when evaluating the atherogenic potential of LDL subfractions and support its use as a superior biomarker for ASCVD risk stratification. Collectively, these observations underscore the importance of characterizing LDL particle properties, particularly size and number, as potential biomarkers of residual cardiovascular risk. Given their susceptibility to oxidative modification, sdLDL particles not only represent a predictive marker but also serve as precursors to oxidized LDL, thereby directly linking them to the pathogenesis of AS (137).

### 7.2 oxLDL

It is a key player in the development of AS and contributes significantly to atherogenicity through various mechanisms. oxLDL promotes atherogenesis by inducing endothelial dysfunction by triggering oxidative stress, which results in the production of ROS and the activation of pro-inflammatory pathways (138). Oxidative modification alters the properties of LDL, making it more prone to uptake by macrophages through scavenger receptors, particularly CD36 and SR-A, leading to the formation of foam cells (138). The accumulation of foam cells within the arterial wall is a hallmark of early atherosclerotic lesions and is associated with chronic inflammation, further perpetuating the cycle of atherogenesis. Moreover, within the arterial wall, oxLDL stimulates the release of pro-inflammatory cytokines such as interleukin-6 (IL-6) and tumor necrosis factor-alpha (TNF-α) from macrophages and vascular SMCs (115). These cytokines recruit additional immune cells to the site of inflammation, amplifying the inflammatory response and contributing to the progression of atherosclerotic plaques (116). Furthermore, oxLDL can upregulate the expression of adhesion molecules, such as VCAM-1 and ICAM-1, on ECs, enhancing monocyte adhesion and migration into the intima, which is essential for the formation of atherosclerotic lesions (117).

In addition to its inflammatory effects, oxLDL, through its reactive lipid species, also interferes with normal lipid metabolism and clearance processes. These oxidative modifications can hinder the ability of LDL to be recognized and cleared by LDLR, leading to an accumulation of LDL particles in the circulation and within the arterial wall (139). The resulting lipid accumulation, combined with the inflammatory response, creates a favorable environment for plaque formation and instability, increasing the risk of CVEs. oxLDL can stimulate the proliferation and migration of SMCs (Figure 1), contributing to the development of fibrous caps over atherosclerotic plaques, which can either stabilize or destabilize the plaque depending on the surrounding conditions (140).

Ultimately, the complex interplay between oxLDL, inflammation, lipid metabolism, and vascular cell dynamics underscores its significant contributions to atherogenicity, making it a critical target for therapeutic intervention in the prevention and management of AS and its related CVDs. The presence of oxLDL particles also holds clinical significance in assessing atherogenic risk (141). Patients with higher concentrations of oxLDL demonstrate a greater risk of plaque rupture and acute coronary events, with elevated oxLDL levels linked to systemic inflammation and AS, highlighting its value in cardiovascular risk assessment protocols (142).

### 7.3 Lp(a)

The structure of Lp(a) allows it to contribute to atherogenicity through multiple mechanisms, including promoting lipid accumulation, fostering inflammation, and interfering with fibrinolysis (143). One key feature of Lp(a) that enhances its atherogenic potential is the presence of oxPLs on its surface, which can induce pro-inflammatory responses in vascular cells, leading to endothelial dysfunction and vascular inflammation (144). These oxPLs can interact with scavenger receptors on macrophages, promoting foam cell formation and plaque development. Furthermore, Lp(a) has been shown to stimulate the production of pro-inflammatory cytokines and adhesion molecules, such as interleukin-8 and VCAM-1, which further enhance monocyte recruitment and retention within the arterial wall (145).

Another contribution of Lp(a) to atherogenesis is its interference with the fibrinolytic system. Lp(a) has an LDL core bound to apo(a), a unique glycoprotein that shares structural homology with plasminogen and competes with it for binding sites on fibrin and ECs, which can inhibit plasmin formation. This impairment of the fibrinolytic pathway can lead to a prothrombotic state and contribute to the development of atherosclerotic plaques with increased thrombotic potential (146). Additionally, Lp(a) can be preferentially retained in the arterial wall due to its high affinity for extracellular matrix components such as proteoglycans, further enhancing lipid deposition and plaque stability (147). Moreover, recent studies have demonstrated that elevated Lp(a) levels, even in individuals with no other lipid abnormalities, are associated with increased arterial wall inflammation and early atherogenesis (148). This suggests that Lp(a) independently contributes to atherogenicity, making it an important biomarker and potential therapeutic target in the context of CVD.

### 7.4 L5/LDL(-)

It has garnered attention in recent years for its significant contributions to atherogenicity. L5/LDL(-) is characterized by its negative charge, which results from the presence of additional apolipoproteins with low isoelectric points (pI) such as apoE (pI 5.5), apoAI (pI 5.4), apoCIII (pI 5.1), and apo(a) (pI 5.5), as well as increased NEFA and specific phospholipids. This unique composition enhances its ability to promote AS through several mechanisms. L5/LDL(-) has increased affinity for proteoglycans of the arterial wall, where it can accumulate and contribute to the formation of atherosclerotic plaques. Moreover, L5/LDL(-) exhibits greater pro-inflammatory potential compared to native LDL particles (149). Studies have shown that L5/LDL(-) can induce EC dysfunction, promoting the expression of adhesion molecules such as ICAM-1 and VCAM-1 (150). This upregulation facilitates the recruitment and retention of monocytes, which differentiate into macrophages and form foam cells, a critical component of atherosclerotic lesions (151). Additionally, L5/LDL(-) can activate signaling pathways that lead to the production of pro-inflammatory cytokines, exacerbating the inflammatory milieu within the arterial wall and perpetuating the atherosclerotic process (104).

Another significant aspect of L5/LDL(-)'s contribution to atherogenicity is its impaired clearance from circulation, which may reflect impaired metabolism and contribute to lipid imbalance. L5/LDL(-) is less efficiently cleared from circulation due to its altered recognition by LDLR, leading to prolonged exposure of vascular tissues to its atherogenic effects. This impaired clearance probably results in higher plasma levels of L5/LDL(-), which are associated with increased cardiovascular risk (100). Furthermore, the accumulation of L5/LDL(-) in the arterial wall can induce oxidative stress and further lipid peroxidation, creating a vicious cycle that exacerbates endothelial dysfunction and promotes plaque instability (151).

The atherogenic effects of L5/LDL(-) have been supported by clinical studies that highlight the correlation between elevated levels of L5/LDL(-) and increased incidence of CVEs. For instance, recent research by Chan et al. showed that elevated concentrations of L5/LDL(-) are associated with a significantly higher risk of CAD (152, 153), independent of other lipid parameters. This evidence underscores the importance of L5/LDL(-) as a critical player in the pathogenesis of AS, suggesting that it may serve as a valuable biomarker and potential therapeutic target in CVD management.

The clinical relevance of LDL particle characteristics in atherogenicity is underscored by their substantial impact on cardiovascular risk assessment and management. The recognition that not all LDL particles have the same characteristics has led to a paradigm shift in how clinicians should approach dyslipidemia, ultimately leading to improved patient outcomes through more personalized therapeutic strategies and better risk stratification.

## 8 Therapeutic approaches targeting LDL particle traits

**Figure 2:**
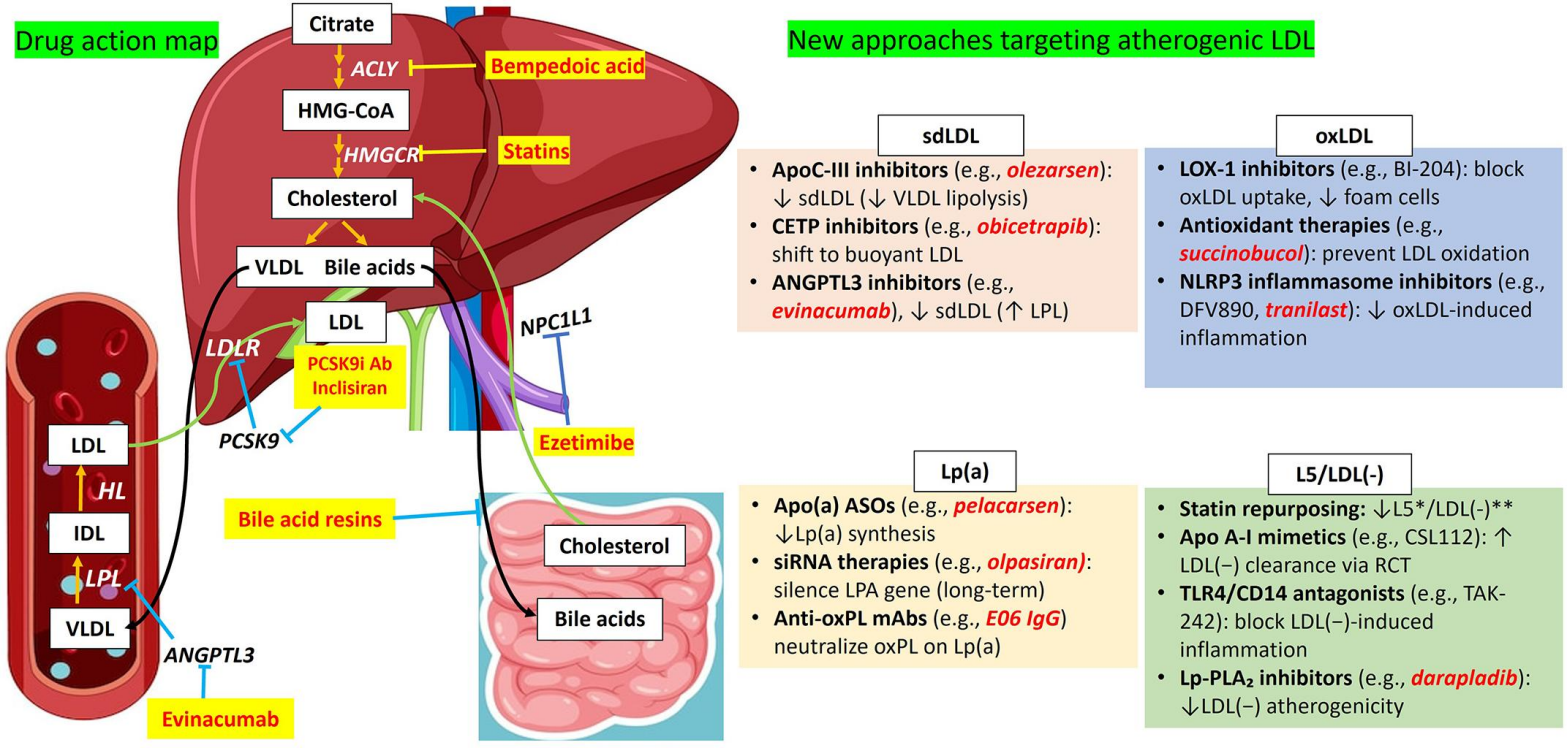
Overview of conventional LDL-C–lowering therapies and emerging strategies targeting atherogenic LDL subfractions. This figure contrasts established lipid-lowering therapies with emerging interventions aimed at specific pathogenic LDL subfractions implicated in atherogenesis, including sdLDL, oxLDL, Lp(a), and L5/LDL(-). Conventional therapies, such as statins, PCSK9 inhibitors, bempedoic acid, and ezetimibe, primarily act through hepatic and intestinal pathways to lower circulating LDL-C levels. In contrast, novel agents, including evinacumab, inclisiran, and experimental therapeutics, are designed to exert systemic effects or selectively modulate the metabolism, clearance, or pathogenicity of distinct LDL subfractions. Together, these strategies reflect a paradigm shift toward mechanistically tailored interventions aimed at optimizing cardiovascular risk reduction beyond general LDL-C lowering. ACLY, ATP citrate lyase; ANGPTL3, angiopoietin-like protein 3; ASOSs, apo(a) antisense oligonucleotides; CETP, cholesteryl ester transfer protein; HL, hepatic lipase; HMGCR, HMG-Coa reductase; IDL, intermediate-density lipoprotein; L5/LDL(-), electronegative LDL; LDL, low-density lipoprotein; LDLR, low-density lipoprotein receptor; Lp(a), lipoprotein(a); LPL, lipoprotein lipase; NPC1l, Niemann-Pick C1-like 1; oxLDL, oxidized LDL; PCK9, protein convertase subtilisin/kexin type 9; PCK9i Ab, proprotein convertase subtilisin/kexin type 9 inhibitor antibody; sdLDL, small dense LDL; VLDL, very low-density lipoprotein.

Therapeutic approaches targeting LDL particle characteristics have evolved significantly in recent years, driven by a growing understanding of the distinct atherogenic potential associated with different LDL subfractions. Traditional lipid-lowering strategies have primarily focused on reducing overall LDL-C levels; however, emerging evidence highlights the necessity of addressing the qualitative characteristics of LDL particles, such as their density, size, and oxidative state (Table 2), to more effectively mitigate cardiovascular risk (Figure 2) (154).

**Table 2:** Conventional lipid-lowering treatments vs. emerging LDL classes-targeted therapies.

Therapy	Mechanism	Limitations	Emerging potential alternative
Statins	HMGCR inhibition	Myopathy and diabetes risk	PCSK9 inhibitors, Inclisiran (PCSK9 siRNA), Bempedoic acid
Bile acid sequestrants	Bind bile acids in the gut	GI side effects, weak LDL reduction	Obeticholic acid (FXR agonist)
Fibrates	Activate PPAR-α	Limited LDL-lowering effect, renal/hepatic toxicity	Olezersans (APOC3 inhibitor), Evinacumab (ANGPTL3 inhibitor), Pemafibrate (PPAR-α modulator)
PCSK9i	LDLR stabilization	High cost, injections	Inclisiran, Oral PCSK9i, gene editing approaches
Ezetimibe	NPC1L1 inhibition	Modest efficacy	Obicetrapib (CETP inhibitors), Oral PCSK9i, Evinacumab, Inclisiran
Bempedoic Acid	ACLY inhibition	Limited sdLDL reduction	Oral PCSK9, ANGPTL3 inhibitors, Inclisiran

ACLY, ATP citrate lyase; ANGPTL3, angiopoietin-like protein 3; CETP, cholesteryl ester transfer protein; GI, gastrointestinal; HMGCR, HMG-CoA reductase; LDL, low-density lipoprotein; LDLR, low-density lipoprotein receptor; NPC1l, Niemann-Pick C1-like 1; PCSK9, protein convertase subtilisin/kexin type 9; PPAR, peroxisome proliferator-activated receptors; sdLDL, small, dense LDL.

The most established therapeutic strategy for lowering LDL-C is the use of statins, which inhibit the enzyme HMG-CoA reductase to reduce cholesterol synthesis in the liver. Statins not only lower overall LDL-C levels but have also been shown to shift the LDL particle distribution toward larger, more buoyant particles, which are considered less atherogenic (155). By doing so, statins can significantly reduce the incidence of CVEs. However, while statins remain the cornerstone of lipid management, their impact on LDL particle size varies among individuals, and not all patients achieve adequate LDL particle modification with statin therapy alone (62). To address the limitations of statin therapy, additional agents to modify lipid levels have been introduced to target specific LDL particle traits. For example, ezetimibe, a cholesterol absorption inhibitor, has been shown to further reduce LDL-C levels when used in conjunction with statins (154). Clinical trials indicate that this combination therapy can lead to a more pronounced decrease in sdLDL particles, potentially enhancing cardiovascular protection (25). In addition, it has been shown that therapies using different statins also decrease the proportion of L5/LDL(-) within total LDL (156–159).

In the context of sdLDL, novel and promising therapies aimed at reducing hypertriglyceridemia, such as inhibitors of the action of apoC-III or angiopoietin-like protein 3, including monoclonal antibodies, antisense oligonucleotides, or small interfering RNA (siRNA), are relevant since lowering TGs is expected to increase LDL particle size (Table 2; Figure 2).

Furthermore, proprotein convertase subtilisin/kexin type 9 (PCSK9) inhibitors have emerged as a novel class of lipid-lowering agents that can dramatically lower LDL-C levels and alter LDL particle composition. PCSK9 inhibitors increase the number of available LDLRs on hepatocytes, promoting the clearance of atherogenic LDL particles from the circulation and thereby decreasing the concentration of sdLDL (160). Recent research has also investigated the potential of novel therapies such as apoB-targeting agents and antisense oligonucleotides to further refine LDL particle traits. These therapies aim to specifically reduce the concentration of apoB-containing lipoproteins, which include LDL and VLDL. Preliminary studies suggest that targeting apoB may lead to significant reductions in both LDL-C and the number of Lp(a) particles, thus lowering atherogenic risk (161). Olpasiran, a siRNA, blocks Lp(a) production by preventing translation of apo(a) mRNA (162, 163). Treatment with the oxLDL-specific antibody orticumab reduced aortic AS by 43%, subvalvular plaque area by 50% and the macrophage content by 31% (164).

In addition to pharmacological therapies, lifestyle modifications play a crucial role in targeting LDL particle traits. Diet, physical activity, and weight management can significantly influence LDL particle size and density (165, 166). Diets rich in omega-3 FAs, soluble fiber, and monounsaturated fats have been shown to favorably modify LDL particle characteristics by promoting larger, less atherogenic particles (167). Regular physical activity not only aids in weight loss but also enhances the lipid profile by increasing the proportion of large, buoyant LDL particles while reducing sdLDL particles (168).

Breakthroughs in personalized medicine and next-generation lipid analysis tools are revolutionizing therapeutic approaches through tailored strategies based on a patient's distinct LDL profile. By utilizing comprehensive lipid profiles, clinicians can identify patients with a predominance of sdLDL particles and implement targeted interventions to address these specific traits effectively (113).

Essentially, the landscape of therapeutic approaches targeting LDL particle traits has expanded significantly in recent years, incorporating a combination of pharmacological interventions, lifestyle modifications, and personalized medicine strategies. By focusing on not only the reduction of LDL-C but also the qualitative characteristics of LDL particles, healthcare providers can enhance the efficacy of cardiovascular risk management and potentially improve patient outcomes in the long term.

## 9 Emerging research directions in understanding LDL particle contributions to atherogenicity

Emerging research efforts to understand the atherogenic contributions of LDL particles are increasingly focused on the complex mechanisms that underlie LDL's role in CVD. Traditional perspectives have primarily emphasized the quantity of circulating LDL-C as a risk factor; however, contemporary investigations are shifting toward a more nuanced understanding of LDL particle characteristics, including density, size, and biochemical modifications, as critical determinants of atherogenic potential. Recent studies have begun to elucidate how these LDL particle traits interact with various biological pathways, influencing the pathogenesis of AS and associated CVEs.

One promising area of research involves the exploration of LDL particle heterogeneity and its implications for vascular inflammation. New approaches utilizing advanced lipidomic profiling techniques aim to quantify and characterize different LDL subfractions in various populations and their respective roles in atherogenesis. By employing NMR spectroscopy and mass spectrometry, researchers can identify distinct LDL subclasses and their relative contributions to cardiovascular risk. This level of understanding could inform strategies that specifically counteract the atherogenic effects of distinct LDL subfractions (113).

Another emerging direction is the investigation of genetic and epigenetic factors that influence LDL particle characteristics. The role of genetic variants, such as those affecting apoB and PCSK9, in modulating LDL particle size and number is actively studied (1). Epigenetic modifications, such as DNA methylation and histone modifications, are also being investigated for their potential to influence LDL metabolism and particle composition, offering new avenues for therapeutic intervention (169). Understanding the genetic underpinnings of LDL particle traits could provide critical insights into individual susceptibility to atherogenicity and may inform personalized approaches to lipid management.

The role of gut microbiota in modulating LDL particle characteristics and their subsequent atherogenic potential represents another exciting research frontier. Emerging evidence suggests that the composition of gut microbiota can impact lipid metabolism, leading to alterations in LDL particle traits (170). Certain gut bacteria have been shown to influence the absorption and processing of dietary lipids, which may subsequently affect LDL particle formation and composition (171). Investigating the microbiome's impact on LDL particle characteristics could unveil novel therapeutic strategies that harness the gut-lipid axis to mitigate cardiovascular risk.

Additionally, the intersection of inflammation, oxidative stress, and LDL particle dynamics is garnering increasing attention. Recent studies have highlighted the role of inflammatory cytokines in modulating LDL particle composition, leading to a greater proportion of sdLDL particles (172). Understanding how systemic inflammation interacts with LDL metabolism may yield insights into the development of new anti-inflammatory therapies aimed at reducing LDL-related atherogenicity. This focus on inflammation aligns with the broader recognition of CVD as an inflammatory condition, necessitating a holistic approach to prevention and treatment.

Finally, the integration of novel imaging techniques, such as intravascular ultrasound and positron emission tomography, is enhancing our ability to visualize LDL particle behavior within the arterial wall and its relationship to plaque formation and stability (173). These technologies can provide real-time assessments of LDL dynamics and their contributions to atherogenesis, preparing the path for more precise risk stratification and management strategies (174). Emerging research directions in understanding LDL particle contributions to atherogenicity are multifaceted, focusing on the intricate interplay of particle characteristics, genetic and epigenetic influences, gut microbiota interactions, inflammation, and advanced imaging technologies. These innovative approaches hold the potential to deepen our understanding of LDL's role in CVD and inform the development of more effective, personalized therapeutic interventions.

## 10 Critical evaluation of L5/LDL(-) as an atherogenic LDL subfraction and emerging biomarker

Among atherogenic LDL subfractions, L5/LDL(-) has gained increasing attention due to its pro-inflammatory, endothelial-damaging, and atherogenic properties demonstrated in experimental settings (31). However, despite compelling *in vitro* and animal model data, the clinical evidence supporting L5/LDL(-) as a diagnostic or prognostic biomarker remains limited and preliminary. To date, the majority of L5/LDL(-) studies have been conducted in relatively small cohorts, often in highly selected populations such as patients with metabolic syndrome, T2DM, STEMI, or chronic autoimmune disorders (32, 89, 96, 99, 100, 152, 153, 159, 175–178). These studies, while suggestive, often lack sufficient power to generalize findings across broader populations. Furthermore, differences in lipoprotein separation techniques, L5/LDL(-) quantification methods, and patient phenotyping limit the reproducibility and comparability of findings across studies. Longitudinal data on L5/LDL(-) levels and cardiovascular outcomes are particularly scarce.

Moreover, many studies have not fully controlled for potential confounding factors such as concurrent lipid-lowering therapies, inflammation, or comorbid conditions that may influence both LDL electronegativity and cardiovascular risk. Importantly, while the association between elevated L5/LDL(-) levels and endothelial dysfunction has been shown mechanistically *in vitro*, direct causal links in human populations remain to be established.

Given these limitations, the role of L5/LDL(-) as a clinical biomarker must be interpreted with caution. The current level of evidence is best characterized as hypothesis-generating rather than conclusive. Future studies with larger, more diverse populations, standardized methods for L5/LDL(-) quantification, and longitudinal follow-up will be critical to validate L5/LDL(-)'s utility as a biomarker and therapeutic target in AS.

## 11 Clinical practice guidelines and emerging relevance of LDL subfractions

Current clinical practice guidelines, including those from the American College of Cardiology (ACC)/American Heart Association (AHA) and the European Society of Cardiology (ESC)/European Atherosclerosis Society (EAS), emphasize the central role of LDL-C in cardiovascular risk assessment and management (5, 179). Treatment strategies focus primarily on lowering total LDL-C through lifestyle modification and pharmacologic therapy, most notably statins, ezetimibe, and PCSK9 inhibitors, based on absolute risk categories and LDL-C thresholds.

Despite mounting evidence that certain LDL subfractions, such as sdLDL, oxLDL, Lp(a), and L5/LDL(-), may be more directly implicated in atherogenesis than total LDL-C, these subfractions are not currently incorporated into routine risk stratification algorithms or treatment guidelines (31). Several reasons underlie this gap, including the lack of standardized, widely available assays for subfraction measurement, limited large-scale clinical data linking subfractions to outcomes independently of LDL-C, and the absence of intervention studies specifically targeting these subfractions (9, 180).

Nevertheless, a growing body of research suggests that patients with normal or mildly elevated LDL-C levels may still carry a high burden of atherogenic LDL subfractions, highlighting the need for more refined lipid profiling in selected populations, such as those with metabolic syndrome, diabetes, or residual cardiovascular risk despite statin therapy (181). Emerging strategies, including lifestyle interventions, niacin, fibrates, and some newer lipid-modifying agents (e.g., angiopoietin-like protein 3 inhibitors), may preferentially impact these atherogenic subfractions, although their clinical roles remain to be fully defined (65, 182, 183).

As such, LDL subfraction analysis currently resides in the realm of research or specialized lipid clinics rather than mainstream clinical practice. Future inclusion in guidelines will likely depend on validation from large-scale, prospective studies demonstrating incremental predictive value and clear benefit from subfraction-targeted therapies.

## 12 Conclusion

This review comprehensively explores the multifaceted nature of atherogenic LDL particles by examining their density, size and electronegativity, and elucidates how these characteristics contribute to their atherogenic potential. Current classification systems provide a framework for distinguishing various LDL subfractions based on their physicochemical properties, yet understanding the underlying mechanisms requires robust methodological approaches for precise measurement and characterization. The evidence indicates that smaller, denser, and more electronegative LDL particles exhibit a higher propensity for contributing to atherogenesis due to enhanced susceptibility to oxidative modification and preferential uptake by macrophages.

Factors such as diet, genetics, and metabolic conditions further modulate LDL particle properties, adding another layer of complexity to their role in CVD risk. The clinical relevance of LDL subfractions and their distinct characteristics suggest that tailored therapeutic strategies targeting these properties may offer a more effective approach to reducing atherogenicity and mitigating CVD risk. This growing body of evidence underscores the importance of integrating these insights into clinical practice to improve risk stratification and therapeutic interventions.

## Glossary

ACLY: ATP citrate lyase
Apo(a): apolipoprotein(a)
ApoA-I: apolipoprotein A-1
ApoB-100: apolipoprotein B100
ApoC-III: apolipoprotein C-III
ApoD: apolipoprotein D
ApoE: apolipoprotein E
ApoF: apolipoprotein F
ApoJ: apolipoprotein J
AS: atherosclerosis
ASOSs: apo(a) antisense oligonucleotides
ASCVD: atherosclerotic cardiovascular disease
CAD: coronary artery disease
CD36: scavenger receptor class B member 3
CE: cholesterol ester
CETP: cholesteryl ester transfer protein
CVD: cardiovascular disease
CVEs: cardiovascular events
DM: diabetes mellitus
eNOS: endothelial nitric oxide synthase
FPLC: fast protein liquid chromatography
HDL: high-density lipoprotein
HDL-C: high-density lipoprotein cholesterol
HL: hepatic lipase
HMGCR: HMG-CoA reductase
ICAM-1: intercellular adhesion molecule-1
IDL: intermediate-density lipoprotein
IL-1β: interleukin-1 beta
L5/LDL(-): electronegative LDL
LDL: low-density lipoprotein
LDLR: low-density lipoprotein receptor
LOX-1: lectin-like oxidized LDL receptor-1
Lp(a): lipoprotein(a)
LPC: lysophosphatidylcholine
LPL: lipoprotein lipase
MMPs: metalloproteinases
NEFA: non-esterified fatty acids
NF-κB: nuclear factor kappa B
NLRP3: nucleotide-binding domain, leucine-rich-containing family, pyrin domain-containing 3
NO: nitric oxide
NPC1l: Niemann-Pick C1-like 1
NMR: nuclear magnetic resonance
oxLDL: oxidized LDL
oxLp(a): oxidized Lp(a)
oxPL: oxidized phospholipids
PCK9: protein convertase subtilisin/kexin type 9
PCK9i Ab: proprotein convertase subtilisin/kexin type 9 inhibitor antibody
pI: isoelectric points
PUFAs: polyunsaturated fatty acids
ROS: reactive oxygen species
RA: rheumatoid arthritis
sdLDL: small dense LDL
siRNA: small interfering RNA
SMCs: smooth muscle cells
SR-A: scavenger receptor class A
STEMI: ST-elevation myocardial infarction
TG: triacylglycerol
TNF-α: tumor necrosis factor-α
T2DM: type 2 diabetes mellitus
VCAM-1: vascular cell adhesion molecule-1
VLDL: very low-density lipoprotein

## References

[1] Boren J Chapman MJ Krauss RM Packard CJ Bentzon JF Binder CJ Low-density lipoproteins cause atherosclerotic cardiovascular disease: pathophysiological, genetic, and therapeutic insights: a consensus statement from the European atherosclerosis society consensus panel. Eur Heart J. (2020) 41(24):2313–30. 10.1093/eurheartj/ehz962 32052833 PMC7308544

[2] Kannel WB Dawber TR Kagan A Revotskie N Stokes J 3rd. Factors of risk in the development of coronary heart disease–six year follow-up experience. The framingham study. Ann Intern Med. (1961) 55:33–50. 10.7326/0003-4819-55-1-33 13751193

[3] Prospective Studies Collaboration, Lewington S Whitlock G Clarke R Sherliker P Emberson J Blood cholesterol and vascular mortality by age, sex, and blood pressure: a meta-analysis of individual data from 61 prospective studies with 55,000 vascular deaths. Lancet. (2007) 370(9602):1829–39. 10.1016/S0140-6736(07)61778-4 18061058

[4] Emerging Risk Factors Collaboration, Di Angelantonio E Gao P Pennells L Kaptoge S Caslake M Lipid-related markers and cardiovascular disease prediction. JAMA. (2012) 307(23):2499–506. 10.1001/jama.2012.6571 22797450 PMC4211641

[5] Visseren FLJ Mach F Smulders YM Carballo D Koskinas KC Bäck M 2021 ESC guidelines on cardiovascular disease prevention in clinical practice. Eur Heart J. (2021) 42(34):3227–337. 10.1093/eurheartj/ehab484 34458905

[6] Grundy SM Stone NJ Bailey AL Beam C Birtcher KK Blumenthal RS 2018 AHA/ACC/AACVPR/AAPA/ABC/ACPM/ADA/AGS/APhA/ASPC/NLA/PCNA guideline on the management of blood cholesterol: a report of the American College of Cardiology/American Heart Association task force on clinical practice guidelines. Circulation. (2019) 139(25):e1082–143. 10.1161/CIR.0000000000000625 30586774 PMC7403606

[7] Rong S Li B Chen L Sun Y Du Y Liu B Association of low-density lipoprotein cholesterol levels with more than 20-year risk of cardiovascular and all-cause mortality in the general population. J Am Heart Assoc. (2022) 11(15):e023690. 10.1161/JAHA.121.023690 35904192 PMC9375485

[8] Errigo A Dore MP Portoghese M Pes GM. The cholesterol paradox in long-livers from a sardinia longevity hot spot (blue zone). Nutrients. (2025) 17(5):765. 10.3390/nu17050765 40077635 PMC11901585

[9] Chary A Tohidi M Hedayati M. Association of LDL-cholesterol subfractions with cardiovascular disorders: a systematic review. BMC Cardiovasc Disord. (2023) 23(1):533. 10.1186/s12872-023-03578-0 37914996 PMC10621218

[10] Sampson UK Fazio S Linton MF. Residual cardiovascular risk despite optimal LDL cholesterol reduction with statins: the evidence, etiology, and therapeutic challenges. Curr Atheroscler Rep. (2012) 14(1):1–10. 10.1007/s11883-011-0219-7 22102062 PMC3697085

[11] Verma KP Inouye M Meikle PJ Nicholls SJ Carrington MJ Marwick TH. New cardiovascular risk assessment techniques for primary prevention: JACC review topic of the week. J Am Coll Cardiol. (2022) 80(4):373–87. 10.1016/j.jacc.2022.05.015 35863853

[12] Balling M Nordestgaard BG Langsted A Varbo A Kamstrup PR Afzal S. Small dense low-density lipoprotein cholesterol predicts atherosclerotic cardiovascular disease in the Copenhagen general population study. J Am Coll Cardiol. (2020) 75(22):2873–5. 10.1016/j.jacc.2020.03.072 32498816

[13] Morel DW Hessler JR Chisolm GM. Low density lipoprotein cytotoxicity induced by free radical peroxidation of lipid. J Lipid Res. (1983) 24(8):1070–6. 10.1016/S0022-2275(20)37921-9 6415194

[14] Rehberger Likozar A Zavrtanik M Sebestjen M. Lipoprotein(a) in atherosclerosis: from pathophysiology to clinical relevance and treatment options. Ann Med. (2020) 52(5):162–77. 10.1080/07853890.2020.1775287 32453609 PMC7877976

[15] Kraler S Sawamura T Harn GY Chen CH Akhmedov A. Editorial: implications of lipids and modified lipoproteins in atherogenesis: from mechanisms towards novel diagnostic and therapeutic targets. Front Cardiovasc Med. (2023) 10:1245716. 10.3389/fcvm.2023.1245716 37554370 PMC10406132

[16] Seidel D Alaupovic P Furman RH. A lipoprotein characterizing obstructive jaundice. I. Method for quantitative separation and identification of lipoproteins in jaundiced subjects. J Clin Invest. (1969) 48(7):1211–23. 10.1172/JCI106085 4978447 PMC322342

[17] Sata T Havel RJ Jones AL. Characterization of subfractions of triglyceride-rich lipoproteins separated by gel chromatography from blood plasma of normolipemic and hyperlipemic humans. J Lipid Res. (1972) 13(6):757–68. 10.1016/S0022-2275(20)39346-9 4345055

[18] Krauss RM Burke DJ. Identification of multiple subclasses of plasma low density lipoproteins in normal humans. J Lipid Res. (1982) 23(1):97–104. 10.1016/S0022-2275(20)38178-5 7057116

[19] Ivanova EA Myasoedova VA Melnichenko AA Grechko AV Orekhov AN. Small dense low-density lipoprotein as biomarker for atherosclerotic diseases. Oxid Med Cell Longev. (2017) 2017:1273042. 10.1155/2017/1273042 28572872 PMC5441126

[20] Packard CJ Boren J Taskinen MR. Causes and consequences of hypertriglyceridemia. Front Endocrinol (Lausanne). (2020) 11:252. 10.3389/fendo.2020.00252 32477261 PMC7239992

[21] Hirano T. Clinical significance of small dense low-density lipoprotein cholesterol measurement in type 2 diabetes. J Diabetes Investig. (2025) 16(3):370–83. 10.1111/jdi.14398 39778086 PMC11871407

[22] Varghese DS Ali BR. Pathological crosstalk between oxidized LDL and ER stress in human diseases: a comprehensive review. Front Cell Dev Biol. (2021) 9:674103. 10.3389/fcell.2021.674103 34124059 PMC8187772

[23] Tribble DL van den Berg JJ Motchnik PA Ames BN Lewis DM Chait A Oxidative susceptibility of low density lipoprotein subfractions is related to their ubiquinol-10 and alpha-tocopherol content. Proc Natl Acad Sci U S A. (1994) 91(3):1183–7. 10.1073/pnas.91.3.1183 8302851 PMC521478

[24] Pizzimenti S Ciamporcero E Daga M Pettazzoni P Arcaro A Cetrangolo G Interaction of aldehydes derived from lipid peroxidation and membrane proteins. Front Physiol. (2013) 4:242. 10.3389/fphys.2013.00242 24027536 PMC3761222

[25] Verhoye E Langlois MR Asklepios I. Circulating oxidized low-density lipoprotein: a biomarker of atherosclerosis and cardiovascular risk? Clin Chem Lab Med. (2009) 47(2):128–37. 10.1515/CCLM.2009.037 19099526

[26] Fernandez-Higuero JA Benito-Vicente A Etxebarria A Milicua JC Ostolaza H Arrondo JL Structural changes induced by acidic pH in human apolipoprotein B-100. Sci Rep. (2016) 6:36324. 10.1038/srep36324 27824107 PMC5099883

[27] Parthasarathy S Raghavamenon A Garelnabi MO Santanam N. Oxidized low-density lipoprotein. Methods Mol Biol. (2010) 610:403–17. 10.1007/978-1-60327-029-8_24 20013192 PMC3315351

[28] Aviram M. Review of human studies on oxidative damage and antioxidant protection related to cardiovascular diseases. Free Radic Res. (2000) 33(Suppl):S85–97. 11191279

[29] Scipione CA Koschinsky ML Boffa MB. Lipoprotein(a) in clinical practice: new perspectives from basic and translational science. Crit Rev Clin Lab Sci. (2018) 55(1):33–54. 10.1080/10408363.2017.1415866 29262744

[30] Lamon-Fava S Marcovina SM Albers JJ Kennedy H DeLuca C White CC Lipoprotein(a) levels, apo(a) isoform size, and coronary heart disease risk in the framingham offspring study. J Lipid Res. (2011) 52(6):1181–7. 10.1194/jlr.M012526 21478162 PMC3090239

[31] Akyol O Yang CY Woodside DG Chiang HH Chen CH Gotto AM. Comparative analysis of atherogenic lipoproteins L5 and Lp(a) in atherosclerotic cardiovascular disease. Curr Atheroscler Rep. (2024) 26(7):317–29. 10.1007/s11883-024-01209-3 38753254 PMC11192678

[32] Sanchez-Quesada JL Perez A Caixas A Ordonmez-Llanos J Carreras G Payes A Electronegative low density lipoprotein subform is increased in patients with short-duration IDDM and is closely related to glycaemic control. Diabetologia. (1996) 39(12):1469–76. 10.1007/s001250050600 8960828

[33] Sanchez-Quesada JL Jorba O Payes A Otal C Serra-Grima R Gonzalez-Sastre F Ascorbic acid inhibits the increase in low-density lipoprotein (LDL) susceptibility to oxidation and the proportion of electronegative LDL induced by intense aerobic exercise. Coron Artery Dis. (1998) 9(5):249–55. 10.1097/00019501-199809050-00002 9710684

[34] Yang CY Raya JL Chen HH Chen CH Abe Y Pownall HJ Isolation, characterization, and functional assessment of oxidatively modified subfractions of circulating low-density lipoproteins. Arterioscler Thromb Vasc Biol. (2003) 23(6):1083–90. 10.1161/01.ATV.0000071350.78872.C4 12689919

[35] Yang CY Chen HH Huang MT Raya JL Yang JH Chen CH Pro-apoptotic low-density lipoprotein subfractions in type II diabetes. Atherosclerosis. (2007) 193(2):283–91. 10.1016/j.atherosclerosis.2006.08.059 17030034

[36] Puig N Rives J Estruch M Aguilera-Simon A Rotllan N Camacho M Presence of ceramidase activity in electronegative LDL. Int J Mol Sci. (2022) 24(1):165. 10.3390/ijms24010165 36613609 PMC9820682

[37] Corda S Laplace C Vicaut E Duranteau J. Rapid reactive oxygen species production by mitochondria in endothelial cells exposed to tumor necrosis factor-alpha is mediated by ceramide. Am J Respir Cell Mol Biol. (2001) 24(6):762–8. 10.1165/ajrcmb.24.6.4228 11415943

[38] Gaggini M Ndreu R Michelucci E Rocchiccioli S Vassalle C. Ceramides as mediators of oxidative stress and inflammation in cardiometabolic disease. Int J Mol Sci. (2022) 23(5):2719. 10.3390/ijms23052719 35269861 PMC8911014

[39] Liu P Zhu W Chen C Yan B Zhu L Chen X The mechanisms of lysophosphatidylcholine in the development of diseases. Life Sci. (2020) 247:117443. 10.1016/j.lfs.2020.117443 32084434

[40] Benitez S Puig N Rives J Sole A Sanchez-Quesada JL. Can electronegative LDL act as a multienzymatic complex? Int J Mol Sci. (2023) 24(8):7074. 10.3390/ijms24087074 37108253 PMC10138509

[41] Bancells C Benitez S Villegas S Jorba O Ordonez-Llanos J Sanchez-Quesada JL. Novel phospholipolytic activities associated with electronegative low-density lipoprotein are involved in increased self-aggregation. Biochemistry. (2008) 47(31):8186–94. 10.1021/bi800537h 18605697

[42] Itabe H Obama T. The oxidized lipoproteins *in vivo*: its diversity and behavior in the human circulation. Int J Mol Sci. (2023) 24(6):5747. 10.3390/ijms24065747 36982815 PMC10053446

[43] Litvinko NM Skorostetskaya LA Gerlovsky DO. The interaction of phospholipase A(2) with oxidized phospholipids at the lipid-water surface with different structural organization. Chem Phys Lipids. (2018) 211:44–51. 10.1016/j.chemphyslip.2017.10.010 29100946

[44] Bancells C Canals F Benitez S Colome N Julve J Ordonez-Llanos J Proteomic analysis of electronegative low-density lipoprotein. J Lipid Res. (2010) 51(12):3508–15. 10.1194/jlr.M009258 20699421 PMC2975723

[45] Ke LY Engler DA Lu J Matsunami RK Chan HC Wang GJ Chemical composition-oriented receptor selectivity of L5, a naturally occurring atherogenic low-density lipoprotein. Pure Appl Chem. (2011) 83(9):1–14. 10.1351/PAC-CON-10-12-07 PMC3816395 24198440

[46] Grainger DJ Reckless J McKilligin E. Apolipoprotein E modulates clearance of apoptotic bodies *in vitro* and *in vivo*, resulting in a systemic proinflammatory state in apolipoprotein E-deficient mice. J Immunol. (2004) 173(10):6366–75. 10.4049/jimmunol.173.10.6366 15528376

[47] Demuth K Myara I Chappey B Vedie B Pech-Amsellem MA Haberland ME A cytotoxic electronegative LDL subfraction is present in human plasma. Arterioscler Thromb Vasc Biol. (1996) 16(6):773–83. 10.1161/01.ATV.16.6.773 8640405

[48] Zheng C Azcutia V Aikawa E Figueiredo JL Croce K Sonoki H Statins suppress apolipoprotein CIII-induced vascular endothelial cell activation and monocyte adhesion. Eur Heart J. (2013) 34(8):615–24. 10.1093/eurheartj/ehs271 22927557 PMC3578265

[49] Georgila K Vyrla D Drakos E. Apolipoprotein A-I (ApoA-I), immunity, inflammation and cancer. Cancers (Basel). (2019) 11(8):1097. 10.3390/cancers11081097 31374929 PMC6721368

[50] Pedrini S Doecke JD Hone E Wang P Thota R Bush AI Plasma high-density lipoprotein cargo is altered in Alzheimer’s disease and is associated with regional brain volume. J Neurochem. (2022) 163(1):53–67. 10.1111/jnc.15681 36000528 PMC9804612

[51] Morton RE Mihna D. Apolipoprotein F concentration, activity, and the properties of LDL controlling ApoF activation in hyperlipidemic plasma. J Lipid Res. (2022) 63(2):100166. 10.1016/j.jlr.2021.100166 35016907 PMC8953654

[52] Higgins JA Graham JM Davies IG. Separation of plasma lipoproteins in self-generated gradients of iodixanol. Methods Mol Med. (2001) 52:37–49. 10.1385/1-59259-073-X:37 21340930

[53] Otvos J. Measurement of triglyceride-rich lipoproteins by nuclear magnetic resonance spectroscopy. Clin Cardiol. (1999) 22(6 Suppl):II21–7. 10.1002/clc.4960221405 10376193 PMC6655988

[54] Fernandez-Cidon B Candas-Estebanez B Ribalta J Rock E Guardiola-Guionnet M Amigo N Precipitated sdLDL: an easy method to estimate LDL particle size. J Clin Lab Anal. (2020) 34(7):e23282. 10.1002/jcla.23282 32198796 PMC7370712

[55] Ito Y Fujimura M Ohta M Hirano T. Development of a homogeneous assay for measurement of small dense LDL cholesterol. Clin Chem. (2011) 57(1):57–65. 10.1373/clinchem.2010.149559 21051530

[56] Ruuth M Lahelma M Luukkonen PK Lorey MB Qadri S Sadevirta S Overfeeding saturated fat increases LDL (low-density lipoprotein) aggregation susceptibility while overfeeding unsaturated fat decreases proteoglycan-binding of lipoproteins. Arterioscler Thromb Vasc Biol. (2021) 41(11):2823–36. 10.1161/ATVBAHA.120.315766 34470478 PMC8545249

[57] Lu J Jiang W Yang JH Chang PY Walterscheid JP Chen HH Electronegative LDL impairs vascular endothelial cell integrity in diabetes by disrupting fibroblast growth factor 2 (FGF2) autoregulation. Diabetes. (2008) 57(1):158–66. 10.2337/db07-1287 17959932

[58] Moreno-Gordaliza E van der Lee SJ Demirkan A van Duijn CM Kuiper J Lindenburg PW A novel method for serum lipoprotein profiling using high performance capillary isotachophoresis. Anal Chim Acta. (2016) 944:57–69. 10.1016/j.aca.2016.09.038 27776640

[59] Hayashi T Koba S Ito Y Hirano T. Method for estimating high sdLDL-C by measuring triglyceride and apolipoprotein B levels. Lipids Health Dis. (2017) 16(1):21. 10.1186/s12944-017-0417-6 28125987 PMC5270205

[60] Warnick GR Knopp RH Fitzpatrick V Branson L. Estimating low-density lipoprotein cholesterol by the friedewald equation is adequate for classifying patients on the basis of nationally recommended cutpoints. Clin Chem. (1990) 36(1):15–9. 10.1093/clinchem/36.1.15 2297909

[61] Krauss RM. Small dense low-density lipoprotein particles: clinically relevant? Curr Opin Lipidol. (2022) 33(3):160–6. 10.1097/MOL.0000000000000824 35276699 PMC9197986

[62] Superko H Garrett B. Small dense LDL: scientific background, clinical relevance, and recent evidence still a risk even with ‘normal’ LDL-C levels. Biomedicines. (2022) 10(4):829. 10.3390/biomedicines10040829 35453579 PMC9025822

[63] Grammer TB Kleber ME Marz W Silbernagel G Siekmeier R Wieland H Low-density lipoprotein particle diameter and mortality: the ludwigshafen risk and cardiovascular health study. Eur Heart J. (2015) 36(1):31–8. 10.1093/eurheartj/ehu055 24569029

[64] Vekic J Zeljkovic A Cicero AFG Janez A Stoian AP Sonmez A Atherosclerosis development and progression: the role of atherogenic small, dense LDL. Medicina (Kaunas). (2022) 58(2):299. 10.3390/medicina58020299 35208622 PMC8877621

[65] Jin X Yang S Lu J Wu M. Small, dense low-density lipoprotein-cholesterol and atherosclerosis: relationship and therapeutic strategies. Front Cardiovasc Med. (2021) 8:804214. 10.3389/fcvm.2021.804214 35224026 PMC8866335

[66] Chancharme L Thérond P Nigon F Lepage S Couturier M Chapman MJ. Cholesteryl ester hydroperoxide lability is a key feature of the oxidative susceptibility of small, dense LDL. Arterioscler Thromb Vasc Biol. (1999) 19(3):810–20. 10.1161/01.ATV.19.3.810 10073990

[67] Lorey MB Oorni K Kovanen PT. Modified lipoproteins induce arterial wall inflammation during atherogenesis. Front Cardiovasc Med. (2022) 9:841545. 10.3389/fcvm.2022.841545 35310965 PMC8927694

[68] Sniderman AD Scantlebury T Cianflone K. Hypertriglyceridemic hyperapob: the unappreciated atherogenic dyslipoproteinemia in type 2 diabetes mellitus. Ann Intern Med. (2001) 135(6):447–59. 10.7326/0003-4819-135-6-200109180-00014 11560458

[69] Chung CP Oeser A Raggi P Sokka T Pincus T Solus JF Lipoprotein subclasses determined by nuclear magnetic resonance spectroscopy and coronary atherosclerosis in patients with rheumatoid arthritis. J Rheumatol. (2010) 37(8):1633–8. 10.3899/jrheum.090639 20516025 PMC2914215

[70] Katsiki N Nikolic D Montalto G Banach M Mikhailidis DP Rizzo M. The role of fibrate treatment in dyslipidemia: an overview. Curr Pharm Des. (2013) 19(17):3124–31. 10.2174/1381612811319170020 23317397

[71] Porter RR Sparks JR Durstine JL Custer SS Thompson RW Wang X. Effect of exercise training on lipoprotein subclass particle concentrations and sizes in older women: results from a randomized controlled trial. Geriatrics (Basel). (2023) 8(6):116. 10.3390/geriatrics8060116 38132487 PMC10742846

[72] Wang J Hu B Kong L Cai H Zhang C. Native, oxidized lipoprotein(a) and lipoprotein(a) immune complex in patients with active and inactive rheumatoid arthritis: plasma concentrations and relationship to inflammation. Clin Chim Acta. (2008) 390(1-2):67–71. 10.1016/j.cca.2007.12.015 18237550

[73] Borba EF Santos RD Bonfa E Vinagre CG Pileggi FJ Cossermelli W Lipoprotein(a) levels in systemic lupus erythematosus. J Rheumatol. (1994) 21(2):220–3. 8182628

[74] Argraves KM Kozarsky KF Fallon JT Harpel PC Strickland DK. The atherogenic lipoprotein lp(a) is internalized and degraded in a process mediated by the VLDL receptor. J Clin Invest. (1997) 100(9):2170–81. 10.1172/JCI119753 9410893 PMC508411

[75] Taleb A Witztum JL Tsimikas S. Oxidized phospholipids on apoB-100-containing lipoproteins: a biomarker predicting cardiovascular disease and cardiovascular events. Biomark Med. (2011) 5(5):673–94. 10.2217/bmm.11.60 22003918 PMC3230643

[76] Wu HD Berglund L Dimayuga C Jones J Sciacca RR Di Tullio MR High lipoprotein(a) levels and small apolipoprotein(a) sizes are associated with endothelial dysfunction in a multiethnic cohort. J Am Coll Cardiol. (2004) 43(10):1828–33. 10.1016/j.jacc.2003.08.066 15145108

[77] Kuklenyik Z Jones JI Gardner MS Schieltz DM Parks BA Toth CA Core lipid, surface lipid and apolipoprotein composition analysis of lipoprotein particles as a function of particle size in one workflow integrating asymmetric flow field-flow fractionation and liquid chromatography-tandem mass spectrometry. PLoS One. (2018) 13(4):e0194797. 10.1371/journal.pone.0194797 29634782 PMC5892890

[78] Sokooti S Flores-Guerrero JL Heerspink HJL Connelly MA Bakker SJL Dullaart RPF. Triglyceride-rich lipoprotein and LDL particle subfractions and their association with incident type 2 diabetes: the PREVEND study. Cardiovasc Diabetol. (2021) 20(1):156. 10.1186/s12933-021-01348-w 34321006 PMC8320057

[79] Garvey WT Kwon S Zheng D Shaughnessy S Wallace P Hutto A Effects of insulin resistance and type 2 diabetes on lipoprotein subclass particle size and concentration determined by nuclear magnetic resonance. Diabetes. (2003) 52(2):453–62. 10.2337/diabetes.52.2.453 12540621

[80] Meikle TG Huynh K Giles C Meikle PJ. Clinical lipidomics: realizing the potential of lipid profiling. J Lipid Res. (2021) 62:100127. 10.1016/j.jlr.2021.100127 34582882 PMC8528718

[81] Chapman MJ Orsoni A Tan R Mellett NA Nguyen A Robillard P LDL subclass lipidomics in atherogenic dyslipidemia: effect of statin therapy on bioactive lipids and dense LDL. J Lipid Res. (2020) 61(6):911–32. 10.1194/jlr.P119000543 32295829 PMC7269759

[82] Ke LY Chan HC Chen CC Chang CF Lu PL Chu CS Increased APOE glycosylation plays a key role in the atherogenicity of L5 low-density lipoprotein. FASEB J. (2020) 34(7):9802–13. 10.1096/fj.202000659R 32501643

[83] Ke LY Law SH Mishra VK Parveen F Chan HC Lu YH Molecular and cellular mechanisms of electronegative lipoproteins in cardiovascular diseases. Biomedicines. (2020) 8(12):550. 10.3390/biomedicines8120550 33260304 PMC7760527

[84] Tertov VV Bittolo-Bon G Sobenin IA Cazzolato G Orekhov AN Avogaro P. Naturally occurring modified low density lipoproteins are similar if not identical: more electronegative and desialylated lipoprotein subfractions. Exp Mol Pathol. (1995) 62(3):166–72. 10.1006/exmp.1995.1018 8612720

[85] Sanchez-Quesada JL Villegas S Ordonez-Llanos J. Electronegative low-density lipoprotein. A link between apolipoprotein B misfolding, lipoprotein aggregation and proteoglycan binding. Curr Opin Lipidol. (2012) 23(5):479–86. 10.1097/MOL.0b013e328357c933 22964994

[86] Parasassi T Bittolo-Bon G Brunelli R Cazzolato G Krasnowska EK Mei G Loss of apoB-100 secondary structure and conformation in hydroperoxide rich, electronegative LDL(-). Free Radic Biol Med. (2001) 31(1):82–9. 10.1016/S0891-5849(01)00555-X 11425493

[87] Benitez S Villegas V Bancells C Jorba O Gonzalez-Sastre F Ordonez-Llanos J Impaired binding affinity of electronegative low-density lipoprotein (LDL) to the LDL receptor is related to nonesterified fatty acids and lysophosphatidylcholine content. Biochemistry. (2004) 43(50):15863–72. 10.1021/bi048825z 15595841

[88] Gaubatz JW Gillard BK Massey JB Hoogeveen RC Huang M Lloyd EE Dynamics of dense electronegative low density lipoproteins and their preferential association with lipoprotein phospholipase A(2). J Lipid Res. (2007) 48(2):348–57. 10.1194/jlr.M600249-JLR200 17102149

[89] Sanchez-Quesada JL Benitez S Otal C Franco M Blanco-Vaca F Ordonez-Llanos J. Density distribution of electronegative LDL in normolipemic and hyperlipemic subjects. J Lipid Res. (2002) 43(5):699–705. 10.1016/S0022-2275(20)30111-5 11971940

[90] Lund-Katz S Laplaud PM Phillips MC Chapman MJ. Apolipoprotein B-100 conformation and particle surface charge in human LDL subspecies: implication for LDL receptor interaction. Biochemistry. (1998) 37(37):12867–74. 10.1021/bi980828m 9737865

[91] Ivanova EA Bobryshev YV Orekhov AN. LDL electronegativity index: a potential novel index for predicting cardiovascular disease. Vasc Health Risk Manag. (2015) 11:525–32. 10.2147/VHRM.S74697 26357481 PMC4559248

[92] Bancells C Benitez S Jauhiainen M Ordonez-Llanos J Kovanen PT Villegas S High binding affinity of electronegative LDL to human aortic proteoglycans depends on its aggregation level. J Lipid Res. (2009) 50(3):446–55. 10.1194/jlr.M800318-JLR200 18952981

[93] Puig N Montolio L Camps-Renom P Navarra L Jimenez-Altayo F Jimenez-Xarrie E Electronegative LDL promotes inflammation and triglyceride accumulation in macrophages. Cells. (2020) 9(3):583. 10.3390/cells9030583 32121518 PMC7140452

[94] Poznyak AV Sukhorukov VN Surkova R Orekhov NA Orekhov AN. Glycation of LDL: AGEs, impact on lipoprotein function, and involvement in atherosclerosis. Front Cardiovasc Med. (2023) 10:1094188. 10.3389/fcvm.2023.1094188 36760567 PMC9904536

[95] Benitez S Sanchez-Quesada JL Ribas V Jorba O Blanco-Vaca F Gonzalez-Sastre F Platelet-activating factor acetylhydrolase is mainly associated with electronegative low-density lipoprotein subfraction. Circulation. (2003) 108(1):92–6. 10.1161/01.CIR.0000072791.40232.8F 12821559

[96] Chang CK Chen PK Lan JL Chang SH Hsieh TY Liao PJ Association of electronegative LDL with macrophage foam cell formation and CD11c expression in rheumatoid arthritis patients. Int J Mol Sci. (2020) 21(16):5883. 10.3390/ijms21165883 32824307 PMC7461586

[97] Hsu JF Chou TC Lu J Chen SH Chen FY Chen CC Low-density lipoprotein electronegativity is a novel cardiometabolic risk factor. PLoS One. (2014) 9(9):e107340. 10.1371/journal.pone.0107340 25203525 PMC4159324

[98] Ke LY Chan HC Chen CC Lu J Marathe GK Chu CS Enhanced sphingomyelinase activity contributes to the apoptotic capacity of electronegative low-density lipoprotein. J Med Chem. (2016) 59(3):1032–40. 10.1021/acs.jmedchem.5b01534 26766134

[99] Chu CS Chan HC Tsai MH Stancel N Lee HC Cheng KH Range of L5 LDL levels in healthy adults and L5’s predictive power in patients with hyperlipidemia or coronary artery disease. Sci Rep. (2018) 8(1):11866. 10.1038/s41598-018-30243-w 30089847 PMC6082876

[100] Shen MY Hsu JF Chen FY Lu J Chang CM Madjid M Combined LDL and VLDL electronegativity correlates with coronary heart disease risk in asymptomatic individuals. J Clin Med. (2019) 8(8):1193. 10.3390/jcm8081193 31404961 PMC6723521

[101] Chen CH Ke LY Chan HC Chu CS Lee AS Lin KD Electronegative low-density lipoprotein of patients with metabolic syndrome induces pathogenesis of aorta through disruption of the stimulated by retinoic acid 6 cascade. J Diabetes Investig. (2020) 11(3):535–44. 10.1111/jdi.13158 31597015 PMC7232312

[102] Chen DY Sawamura T Dixon RAF Sánchez-Quesada JL Chen CH. Autoimmune rheumatic diseases: an update on the role of atherogenic electronegative LDL and potential therapeutic strategies. J Clin Med. (2021) 10(9):1992. 10.3390/jcm10091992 34066436 PMC8124242

[103] Lee AS Wang YC Chang SS Lo PH Chang CM Lu J Detection of a high ratio of soluble to membrane-bound LOX-1 in aspirated coronary thrombi from patients with ST-segment-elevation myocardial infarction. J Am Heart Assoc. (2020) 9(2):e014008. 10.1161/JAHA.119.014008 31928155 PMC7033847

[104] Chu CS Law SH Lenzen D Tan YH Weng SF Ito E Clinical significance of electronegative low-density lipoprotein cholesterol in atherothrombosis. Biomedicines. (2020) 8(8):254. 10.3390/biomedicines8080254 32751498 PMC7460408

[105] Krauss RM. Lipoprotein subfractions and cardiovascular disease risk. Curr Opin Lipidol. (2010) 21(4):305–11. 10.1097/MOL.0b013e32833b7756 20531184

[106] Musunuru K. Atherogenic dyslipidemia: cardiovascular risk and dietary intervention. Lipids. (2010) 45(10):907–14. 10.1007/s11745-010-3408-1 20524075 PMC2950930

[107] Tabas I Williams KJ Boren J. Subendothelial lipoprotein retention as the initiating process in atherosclerosis: update and therapeutic implications. Circulation. (2007) 116(16):1832–44. 10.1161/CIRCULATIONAHA.106.676890 17938300

[108] Teslovich TM Musunuru K Smith AV Edmondson AC Stylianou IM Koseki M Biological, clinical and population relevance of 95 loci for blood lipids. Nature. (2010) 466(7307):707–13. 10.1038/nature09270 20686565 PMC3039276

[109] Thanassoulis G Campbell CY Owens DS Smith JG Smith AV Peloso GM Genetic associations with valvular calcification and aortic stenosis. N Engl J Med. (2013) 368(6):503–12. 10.1056/NEJMoa1109034 23388002 PMC3766627

[110] Hodgkinson CP Laxton RC Patel K Ye S. Advanced glycation end-product of low density lipoprotein activates the toll-like 4 receptor pathway implications for diabetic atherosclerosis. Arterioscler Thromb Vasc Biol. (2008) 28(12):2275–81. 10.1161/ATVBAHA.108.175992 18818414

[111] DiNicolantonio JJ O'Keefe JH. Effects of dietary fats on blood lipids: a review of direct comparison trials. Open Heart. (2018) 5(2):e000871. 10.1136/openhrt-2018-000871 30094038 PMC6074619

[112] Gallucci G Tartarone A Lerose R Lalinga AV Capobianco AM. Cardiovascular risk of smoking and benefits of smoking cessation. J Thorac Dis. (2020) 12(7):3866–76. 10.21037/jtd.2020.02.47 32802468 PMC7399440

[113] Stanciulescu LA Scafa-Udriste A Dorobantu M. Exploring the association between low-density lipoprotein subfractions and major adverse cardiovascular outcomes-a comprehensive review. Int J Mol Sci. (2023) 24(7):6669. 10.3390/ijms24076669 37047642 PMC10095470

[114] Lin PK Davis GE. Extracellular matrix remodeling in vascular disease: defining its regulators and pathological influence. Arterioscler Thromb Vasc Biol. (2023) 43(9):1599–616. 10.1161/ATVBAHA.123.318237 37409533 PMC10527588

[115] Amin MN Siddiqui SA Ibrahim M Hakim ML Ahammed MS Kabir A Inflammatory cytokines in the pathogenesis of cardiovascular disease and cancer. SAGE Open Med. (2020) 8:2050312120965752. 10.1177/2050312120965752 33194199 PMC7594225

[116] Hong CG Florida E Li H Parel PM Mehta NN Sorokin AV. Oxidized low-density lipoprotein associates with cardiovascular disease by a vicious cycle of atherosclerosis and inflammation: a systematic review and meta-analysis. Front Cardiovasc Med. (2022) 9:1023651. 10.3389/fcvm.2022.1023651 36727024 PMC9885196

[117] Maiolino G Rossitto G Caielli P Bisogni V Rossi GP Calo LA. The role of oxidized low-density lipoproteins in atherosclerosis: the myths and the facts. Mediators Inflamm. (2013) 2013:714653. 10.1155/2013/714653 24222937 PMC3816061

[118] Schaefer EJ Ikezaki H Diffenderfer MR Lim E Liu CT Hoogeveen RC Atherosclerotic cardiovascular disease risk and small dense low-density lipoprotein cholesterol in men, women, African Americans and non-African Americans: the pooling project. Atherosclerosis. (2023) 367:15–23. 10.1016/j.atherosclerosis.2023.01.015 36724690

[119] Thongtang N Diffenderfer MR Ooi EMM Barrett PHR Turner SM Le NA Metabolism and proteomics of large and small dense LDL in combined hyperlipidemia: effects of rosuvastatin. J Lipid Res. (2017) 58(7):1315–24. 10.1194/jlr.M073882 28392500 PMC5496030

[120] Kanonidou C. Small dense low-density lipoprotein: analytical review. Clin Chim Acta. (2021) 520:172–8. 10.1016/j.cca.2021.06.012 34118239

[121] Reimund M Dearborn AD Graziano G Lei H Ciancone AM Kumar A Structure of apolipoprotein B100 bound to the low-density lipoprotein receptor. Nature. (2025) 638(8051):829–35. 10.1038/s41586-024-08223-0 39663455

[122] Castillo-Nunez Y Morales-Villegas E Aguilar-Salinas CA. Triglyceride-rich lipoproteins: their role in atherosclerosis. Rev Invest Clin. (2022) 74(2):061–70. 10.24875/RIC.21000416 34759386

[123] Bjornheden T Babyi A Bondjers G Wiklund O. Accumulation of lipoprotein fractions and subfractions in the arterial wall, determined in an *in vitro* perfusion system. Atherosclerosis. (1996) 123(1-2):43–56. 10.1016/0021-9150(95)05770-6 8782836

[124] La Belle M Krauss RM. Differences in carbohydrate content of low density lipoproteins associated with low density lipoprotein subclass patterns. J Lipid Res. (1990) 31(9):1577–88. 10.1016/S0022-2275(20)42342-9 2246611

[125] Camejo G Lopez A Lopez F Quinones J. Interaction of low density lipoproteins with arterial proteoglycans. The role of charge and sialic acid content. Atherosclerosis. (1985) 55(1):93–105. 10.1016/0021-9150(85)90169-8 4004985

[126] Jaakkola O Solakivi T Tertov VV Orekhov AN Miettinen TA Nikkari T. Characteristics of low-density lipoprotein subfractions from patients with coronary artery disease. Coron Artery Dis. (1993) 4(4):379–85. 10.1097/00019501-199304000-00010 8261211

[127] de Graaf J Swinkels DW Demacker PN de Haan AF Stalenhoef AF. Differences in the low density lipoprotein subfraction profile between oral contraceptive users and controls. J Clin Endocrinol Metab. (1993) 76(1):197–202. 10.1210/jcem.76.1.8421088 8421088

[128] Chait A Brazg RL Tribble DL Krauss RM. Susceptibility of small, dense, low-density lipoproteins to oxidative modification in subjects with the atherogenic lipoprotein phenotype, pattern B. Am J Med. (1993) 94(4):350–6. 10.1016/0002-9343(93)90144-E 8475928

[129] Dejager S Bruckert E Chapman MJ. Dense low density lipoprotein subspecies with diminished oxidative resistance predominate in combined hyperlipidemia. J Lipid Res. (1993) 34(2):295–308. 10.1016/S0022-2275(20)40756-4 8429263

[130] Tribble DL Holl LG Wood PD Krauss RM. Variations in oxidative susceptibility among six low density lipoprotein subfractions of differing density and particle size. Atherosclerosis. (1992) 93(3):189–99. 10.1016/0021-9150(92)90255-F 1590824

[131] de Graaf J Hak-Lemmers HL Hectors MP Demacker PN Hendriks JC Stalenhoef AF. Enhanced susceptibility to *in vitro* oxidation of the dense low density lipoprotein subfraction in healthy subjects. Arterioscler Thromb. (1991) 11(2):298–306. 10.1161/01.ATV.11.2.298 1998647

[132] Otvos JD Mora S Shalaurova I Greenland P Mackey RH Goff DC Jr. Clinical implications of discordance between low-density lipoprotein cholesterol and particle number. J Clin Lipidol. (2011) 5(2):105–13. 10.1016/j.jacl.2011.02.001 21392724 PMC3070150

[133] Krauss RM. Lipids and lipoproteins in patients with type 2 diabetes. Diabetes Care. (2004) 27(6):1496–504. 10.2337/diacare.27.6.1496 15161808

[134] Hoogeveen RC Gaubatz JW Sun W Dodge RC Crosby JR Jiang J Small dense low-density lipoprotein-cholesterol concentrations predict risk for coronary heart disease: the atherosclerosis risk in communities (ARIC) study. Arterioscler Thromb Vasc Biol. (2014) 34(5):1069–77. 10.1161/ATVBAHA.114.303284 24558110 PMC3999643

[135] Tsai MY Steffen BT Guan W McClelland RL Warnick R McConnell J New automated assay of small dense low-density lipoprotein cholesterol identifies risk of coronary heart disease: the multi-ethnic study of atherosclerosis. Arterioscler Thromb Vasc Biol. (2014) 34(1):196–201. 10.1161/ATVBAHA.113.302401 24233487 PMC4211254

[136] Zubiran R Sampson M Wolska A Remaley AT. Estimated small, dense LDL cholesterol and atherosclerotic cardiovascular risk in the UK biobank. Arterioscler Thromb Vasc Biol. (2025) 45(10):e512–22. 10.1161/ATVBAHA.125.323157 40808652 PMC12369564

[137] Brosolo G Da Porto A Bulfone L Vacca A Bertin N Colussi G Plasma lipoprotein(a) levels as determinants of arterial stiffening in hypertension. Biomedicines. (2021) 9(11):1510. 10.3390/biomedicines9111510 34829739 PMC8615029

[138] Jiang H Zhou Y Nabavi SM Sahebkar A Little PJ Xu S Mechanisms of oxidized LDL-mediated endothelial dysfunction and its consequences for the development of atherosclerosis. Front Cardiovasc Med. (2022) 9:925923. 10.3389/fcvm.2022.925923 35722128 PMC9199460

[139] Kong P Cui ZY Huang XF Zhang DD Guo RJ Han M. Inflammation and atherosclerosis: signaling pathways and therapeutic intervention. Signal Transduct Target Ther. (2022) 7(1):131. 10.1038/s41392-022-00955-7 35459215 PMC9033871

[140] Liu J Ren Y Kang L Zhang L. Oxidized low-density lipoprotein increases the proliferation and migration of human coronary artery smooth muscle cells through the upregulation of osteopontin. Int J Mol Med. (2014) 33(5):1341–7. 10.3892/ijmm.2014.1681 24590381

[141] Poznyak AV Nikiforov NG Markin AM Kashirskikh DA Myasoedova VA Gerasimova EV Overview of OxLDL and its impact on cardiovascular health: focus on atherosclerosis. Front Pharmacol. (2020) 11:613780. 10.3389/fphar.2020.613780 33510639 PMC7836017

[142] Trpkovic A Resanovic I Stanimirovic J Radak D Mousa SA Cenic-Milosevic D Oxidized low-density lipoprotein as a biomarker of cardiovascular diseases. Crit Rev Clin Lab Sci. (2015) 52(2):70–85. 10.3109/10408363.2014.992063 25537066

[143] Dzobo KE Kraaijenhof JM Stroes ESG Nurmohamed NS Kroon J. Lipoprotein(a): an underestimated inflammatory mastermind. Atherosclerosis. (2022) 349:101–9. 10.1016/j.atherosclerosis.2022.04.004 35606070

[144] Tsimikas S. Potential causality and emerging medical therapies for lipoprotein(a) and its associated oxidized phospholipids in calcific aortic valve stenosis. Circ Res. (2019) 124(3):405–15. 10.1161/CIRCRESAHA.118.313864 30702993 PMC6361547

[145] Scipione CA Sayegh SE Romagnuolo R Tsimikas S Marcovina SM Boffa MB Mechanistic insights into lp(a)-induced IL-8 expression: a role for oxidized phospholipid modification of apo(a). J Lipid Res. (2015) 56(12):2273–85. 10.1194/jlr.M060210 26474593 PMC4655984

[146] Dentali F Gessi V Marcucci R Gianni M Grandi AM Franchini M. Lipoprotein(a) as a risk factor for venous thromboembolism: a systematic review and meta-analysis of the literature. Semin Thromb Hemost. (2017) 43(6):614–20. 10.1055/s-0036-1598002 28346964

[147] Boffa MB Koschinsky ML. Oxidized phospholipids as a unifying theory for lipoprotein(a) and cardiovascular disease. Nat Rev Cardiol. (2019) 16(5):305–18. 10.1038/s41569-018-0153-2 30675027

[148] Di Fusco SA Maggioni AP Scicchitano P Zuin M D'Elia E Colivicchi F. Lipoprotein (a), inflammation, and atherosclerosis. J Clin Med. (2023) 12(7):2529. 10.3390/jcm12072529 37048611 PMC10095203

[149] De Castellarnau C Sanchez-Quesada JL Benitez S Rosa R Caveda L Vila L Ordonez-Llanos J. Electronegative LDL from normolipemic subjects induces IL-8 and monocyte chemotactic protein secretion by human endothelial cells. Arterioscler Thromb Vasc Biol. (2000) 20(10):2281–7. 10.1161/01.ATV.20.10.2281 11031216

[150] Abe Y Fornage M Yang CY Bui-Thanh NA Wise V Chen HH L5, the most electronegative subfraction of plasma LDL, induces endothelial vascular cell adhesion molecule 1 and CXC chemokines, which mediate mononuclear leukocyte adhesion. Atherosclerosis. (2007) 192(1):56–66. 10.1016/j.atherosclerosis.2006.06.012 17022986

[151] Lee AS Wang GJ Chan HC Chen FY Chang CM Yang CY Electronegative low-density lipoprotein induces cardiomyocyte apoptosis indirectly through endothelial cell-released chemokines. Apoptosis. (2012) 17(9):1009–18. 10.1007/s10495-012-0726-1 22562555

[152] Chan HC Ke LY Chu CS Lee AS Shen MY Cruz MA Highly electronegative LDL from patients with ST-elevation myocardial infarction triggers platelet activation and aggregation. Blood. (2013) 122(22):3632–41. 10.1182/blood-2013-05-504639 24030386 PMC3837512

[153] Chang PY Chen YJ Chang FH Lu J Huang WH Yang TC Aspirin protects human coronary artery endothelial cells against atherogenic electronegative LDL via an epigenetic mechanism: a novel cytoprotective role of aspirin in acute myocardial infarction. Cardiovasc Res. (2013) 99(1):137–45. 10.1093/cvr/cvt062 23519265

[154] Chait A Eckel RH Vrablik M Zambon A. Lipid-lowering in diabetes: an update. Atherosclerosis. (2024) 394:117313. 10.1016/j.atherosclerosis.2023.117313 37945448

[155] Mudd JO Borlaug BA Johnston PV Kral BG Rouf R Blumenthal RS Beyond low-density lipoprotein cholesterol: defining the role of low-density lipoprotein heterogeneity in coronary artery disease. J Am Coll Cardiol. (2007) 50(18):1735–41. 10.1016/j.jacc.2007.07.045 17964036

[156] Benitez S Ordonez-Llanos J Franco M Marin C Paz E Lopez-Miranda J Effect of simvastatin in familial hypercholesterolemia on the affinity of electronegative low-density lipoprotein subfractions to the low-density lipoprotein receptor. Am J Cardiol. (2004) 93(4):414–20. 10.1016/j.amjcard.2003.10.034 14969613

[157] Pereira EC Bertolami MC Faludi AA Sevanian A Abdalla DS. Antioxidant effect of simvastatin is not enhanced by its association with alpha-tocopherol in hypercholesterolemic patients. Free Radic Biol Med. (2004) 37(9):1440–8. 10.1016/j.freeradbiomed.2004.07.019 15454283

[158] Zhang B Miura S Yanagi D Noda K Nishikawa H Matsunaga A Reduction of charge-modified LDL by statin therapy in patients with CHD or CHD risk factors and elevated LDL-C levels: the SPECIAL study. Atherosclerosis. (2008) 201(2):353–9. 10.1016/j.atherosclerosis.2008.02.024 18395728

[159] Chu CS Ke LY Chan HC Chan HC Chen CC Cheng KH Four statin benefit groups defined by the 2013 ACC/AHA new cholesterol guideline are characterized by increased plasma level of electronegative low-density lipoprotein. Acta Cardiol Sin. (2016) 32(6):667–75. 10.6515/acs20151228e 27899853 PMC5126444

[160] Sabatine MS Giugliano RP Wiviott SD Raal FJ Blom DJ Robinson J Efficacy and safety of evolocumab in reducing lipids and cardiovascular events. N Engl J Med. (2015) 372(16):1500–9. 10.1056/NEJMoa1500858 25773607

[161] Tsimikas S. A test in context: lipoprotein(a): diagnosis, prognosis, controversies, and emerging therapies. J Am Coll Cardiol. (2017) 69(6):692–711. 10.1016/j.jacc.2016.11.042 28183512

[162] O'Donoghue ML Rosenson RS Lopez JAG Lepor NE Baum SJ Stout E The off-treatment effects of olpasiran on lipoprotein(a) lowering: OCEAN(a)-DOSE extension period results. J Am Coll Cardiol. (2024) 84(9):790–7. 10.1016/j.jacc.2024.05.058 39168564

[163] O'Donoghue ML Rosenson RS Gencer B Lopez JAG Lepor NE Baum SJ Small interfering RNA to reduce lipoprotein(a) in cardiovascular disease. N Engl J Med. (2022) 387(20):1855–64. 10.1056/NEJMoa2211023 36342163

[164] Yao Mattisson I Rattik S Bjorkbacka H Ljungcrantz I Terrinoni M Lebens M Immune responses against oxidized LDL as possible targets for prevention of atherosclerosis in systemic lupus erythematosus. Vascul Pharmacol. (2021) 140:106863. 10.1016/j.vph.2021.106863 33857652

[165] Froyen E. The effects of fat consumption on low-density lipoprotein particle size in healthy individuals: a narrative review. Lipids Health Dis. (2021) 20(1):86. 10.1186/s12944-021-01501-0 34362390 PMC8348839

[166] Machado VA Santisteban ARN Martins CM Damasceno NRT Fonseca FA Neto AMF Effects of phytosterol supplementation on lipoprotein subfractions and LDL particle quality. Sci Rep. (2024) 14(1):11108. 10.1038/s41598-024-61897-4 38750162 PMC11096344

[167] Yu E Malik VS Hu FB. Cardiovascular disease prevention by diet modification: JACC health promotion series. J Am Coll Cardiol. (2018) 72(8):914–26. 10.1016/j.jacc.2018.02.085 30115231 PMC6100800

[168] Wang Y Xu D. Effects of aerobic exercise on lipids and lipoproteins. Lipids Health Dis. (2017) 16(1):132. 10.1186/s12944-017-0515-5 28679436 PMC5498979

[169] Ferrari A Fiorino E Giudici M Gilardi F Galmozzi A Mitro N Linking epigenetics to lipid metabolism: focus on histone deacetylases. Mol Membr Biol. (2012) 29(7):257–66. 10.3109/09687688.2012.729094 23095054

[170] Yu Y Raka F Adeli K. The role of the gut microbiota in lipid and lipoprotein metabolism. J Clin Med. (2019) 8(12):2227. 10.3390/jcm8122227 31861086 PMC6947520

[171] Sanchez-Rodriguez E Egea-Zorrilla A Plaza-Diaz J Aragon-Vela J Munoz-Quezada S Tercedor-Sanchez L The gut microbiota and its implication in the development of atherosclerosis and related cardiovascular diseases. Nutrients. (2020) 12(3):605. 10.3390/nu12030605 32110880 PMC7146472

[172] Henein MY Vancheri S Longo G Vancheri F. The role of inflammation in cardiovascular disease. Int J Mol Sci. (2022) 23(21):12906. 10.3390/ijms232112906 36361701 PMC9658900

[173] Wildgruber M Swirski FK Zernecke A. Molecular imaging of inflammation in atherosclerosis. Theranostics. (2013) 3(11):865–84. 10.7150/thno.5771 24312156 PMC3841337

[174] Syed MB Fletcher AJ Forsythe RO Kaczynski J Newby DE Dweck MR Emerging techniques in atherosclerosis imaging. Br J Radiol. (2019) 92(1103):20180309. 10.1259/bjr.20180309 31502858 PMC6849665

[175] Puig N Camps-Renom P Sole A Aguilera-Simon A Jimenez-Xarrie E Fernandez-Leon A Electronegative LDL is associated with plaque vulnerability in patients with ischemic stroke and carotid atherosclerosis. Antioxidants (Basel). (2023) 12(2):438. 10.3390/antiox12020438 36829998 PMC9952764

[176] Estruch M Minambres I Sanchez-Quesada JL Soler M Perez A Ordonez-Llanos J Increased inflammatory effect of electronegative LDL and decreased protection by HDL in type 2 diabetic patients. Atherosclerosis. (2017) 265:292–8. 10.1016/j.atherosclerosis.2017.07.015 28734591

[177] Gambino R Pisu E Pagano G Cassader M. Low-density lipoproteins are more electronegatively charged in type 1 than in type 2 diabetes mellitus. Lipids. (2006) 41(6):529–33. 10.1007/s11745-006-5001-1 16981430

[178] Yano M Inoue M Maehata E Shiba T Yamakado M Hirabayashi Y Increased electronegative charge of serum low-density lipoprotein in patients with diabetes mellitus. Clin Chim Acta. (2004) 340(1-2):93–8. 10.1016/j.cccn.2003.09.020 14734200

[179] Virani SS Newby LK Arnold SV Bittner V Brewer LC Demeter SH 2023 AHA/ACC/ACCP/ASPC/NLA/PCNA guideline for the management of patients with chronic coronary disease: a report of the American Heart Association/American College of Cardiology joint committee on clinical practice guidelines. Circulation. (2023) 148(9):e9–e119. 10.1161/CIR.0000000000001168 37471501

[180] Qiao YN Zou YL Guo SD. Low-density lipoprotein particles in atherosclerosis. Front Physiol. (2022) 13:931931. 10.3389/fphys.2022.931931 36111155 PMC9468243

[181] Razavi AC Bazzano LA He J Krousel-Wood M Dorans KS Razavi MA Discordantly normal ApoB relative to elevated LDL-C in persons with metabolic disorders: a marker of atherogenic heterogeneity. Am J Prev Cardiol. (2021) 7:100190. 10.1016/j.ajpc.2021.100190 34611635 PMC8387299

[182] Gaudet D Karwatowska-Prokopczuk E Baum SJ Hurh E Kingsbury J Bartlett VJ Vupanorsen, an N-acetyl galactosamine-conjugated antisense drug to ANGPTL3 mRNA, lowers triglycerides and atherogenic lipoproteins in patients with diabetes, hepatic steatosis, and hypertriglyceridaemia. Eur Heart J. (2020) 41(40):3936–45. 10.1093/eurheartj/ehaa689 32860031 PMC7750927

[183] Fogacci F Di Micoli V Sabouret P Giovannini M Cicero AFG. Lifestyle and lipoprotein(a) levels: does a specific counseling make sense? J Clin Med. (2024) 13(3):751. 10.3390/jcm13030751 38337445 PMC10856708

